# Culturally Tailored Diabetes Self-Management Education and Support Programs in Black African and Caribbean Adults With Type 2 Diabetes (HEAL-D): Protocol for a Multicenter, Pragmatic Randomized Controlled Trial

**DOI:** 10.2196/71861

**Published:** 2025-09-30

**Authors:** Louise M Goff, Drusus A Johnson, Vicky Bell, Susan Blyden, Peter Bower, Jeremy Dale, Tess Harris, Andrew Healey, Eleanor Hoverd, Huajie Jin, Tony Kelly, Carol Rivas, Clare Robinson, Jayne Thorpe, Sandra Tomlinson, Michael Ussher, Charlotte Wahlich, Barbara McGowan

**Affiliations:** 1 Diabetes Research Centre University of Leicester Leicester United Kingdom; 2 School of Health Sciences University of Manchester Manchester United Kingdom; 3 Unit of Academic Primary Care University of Warwick Coventry United Kingdom; 4 Population Health Research Institute City St George's, University of London London United Kingdom; 5 King’s Health Economics King's College London London United Kingdom; 6 Public Citizen Birmingham United Kingdom; 7 Social Research Institute University College London London United Kingdom; 8 Pragmatic Clinical Trials Unit Queen Mary University of London London United Kingdom; 9 South West London Integrated Care Board London United Kingdom; 10 Public Citizen London United Kingdom; 11 University of Stirling Stirling United Kingdom; 12 Diabetes & Endocrinology Guy's & St Thomas' NHS Foundation Trust London United Kingdom

**Keywords:** type 2 diabetes, education, ethnicity, self-management, clinical trial, protocol

## Abstract

**Background:**

People of Black African and Black Caribbean ethnicity experience higher rates and poorer outcomes of type 2 diabetes (T2D) than people of White European ethnicity; these inequalities are compounded by poor healthcare access. Cultural tailoring of diabetes self-management education and support (DSMES) programs has the potential to improve healthcare engagement and clinical outcomes for ethnic minority groups. Healthy Eating & Active Lifestyles for Diabetes (HEAL-D) is a co-designed, culturally tailored group-based DSMES program for adults of Black African and Black Caribbean ethnicity.

**Objective:**

This trial aims to evaluate the clinical and cost effectiveness of the HEAL-D intervention, compared to standard DSMES programs, in Black African and Black Caribbean adults living with T2D.

**Methods:**

A 24-month, multicenter, pragmatic, open-label, 2-arm, parallel-group, individually randomized group treatment trial will be conducted, with primary end point (glycated hemoglobin [HbA_1c_]) assessment at 12 months. Black African and Black Caribbean adults with T2D (n=300), recruited from 3 to 5 centers in the United Kingdom (including London, West Midlands, and Greater Manchester), will be randomized in a 1:1 ratio to HEAL-D (intervention) or a standard DSMES program (control). HbA_1c_, blood lipids, anthropometric outcomes, blood pressure, physical activity, and patient-reported outcome measures relating to psychological well-being and self-management support, lifestyle behaviors, and health economics will be collected at baseline and follow-up visits (6, 12, and 24 months). Cost-effectiveness will be assessed through a cost-utility analysis conducted from a health and social care perspective. A mixed methods process evaluation will provide a formative evaluation of delivery, intervention fidelity, and implementation of HEAL-D, and an embedded study within a project will assess the impact of multiple long-term conditions on uptake of, and engagement with HEAL-D, and the impact of HEAL-D on multiple long-term conditions. The trial received Research Authority and Research Ethics Council approval on April 22, 2024.

**Results:**

Funding began in August 2023. Site “green light” was received on August 15, 2024, for London; November 29, 2024, for Manchester; and January 31, 2025, for the West Midlands. Recruitment commenced in August 2024 and is due to run for 11 months. As of March 26, 2025, a total of 76 participants have consented. Last patient, last visit is expected in June 2027; primary data analysis is expected to begin in July 2027. Final results are anticipated to be available in September 2027, and publication is expected by the end of 2027.

**Conclusions:**

The HEAL-D trial will address whether a culturally tailored DSMES program, provided in-person or via videoconferencing, is clinically and cost-effective compared to standard DSMES at improving diabetes management in Black African and Black Caribbean adults. If effective, this would provide an evidence-based model of equitable DSMES services and improve the implementation of healthcare programs for ethnic minority groups.

**Trial Registration:**

ISRCTN 1434448; https://www.isrctn.com/ISRCTN14344948

**International Registered Report Identifier (IRRID):**

DERR1-10.2196/71861

## Introduction

### Background

Type 2 diabetes (T2D) prevalence has grown steadily over recent decades, now estimated to affect over 6% of the world’s population [1,2]. T2D is characterized by elevated blood glucose concentrations and increased risk of macrovascular and microvascular complications [3,4]. Such risks culminate in poorer physical and mental health [5], quality of life (QoL) [6], and earlier death [4], with diabetes consuming around 10% of the annual healthcare budget [7].

The effects of T2D are experienced disproportionally by people of Black African and Black Caribbean ethnicity compared to White European ethnicity [8,9], with 2-4 times greater prevalence [8,9], younger onset [10], poorer outcomes for those who are affected, and greater medication requirements [11-13]. A lack of access to T2D healthcare and reduced effectiveness of current T2D treatment programs in Black African and Black Caribbean populations have been proposed as key causes for these inequalities [13-15]. Therefore, the modification of existing T2D treatment pathways may facilitate better adoption and lower attrition rates in those of Black African and Black Caribbean ethnicity [16,17], who make up the second largest and fastest growing UK ethnic minority group [18].

Optimizing diet and physical activity and promoting self-management are integral parts of T2D management [19], with management guidelines recommending attendance at a diabetes self-management education and support (DSMES) program [19]. In the United Kingdom, quality standards require that DSMES programs have an evidence-based curriculum, delivered by trained and competent educators, usually offering at least 6 hours of education [19]. Several DSMES programs are accredited and commissioned [20,21], mainly using a group-based format and face-to-face (F2F) delivery, although digital programs and digital adaptations of F2F programs have been evaluated or implemented following the COVID-19 pandemic [22,23].

Whilst DSMES programs are effective for improving T2D management and cardiovascular, behavioral, and psychological outcomes, they are substantially less effective in people of Black African and Black Caribbean ethnicity [13,16,17]. Despite recommendations for programs to meet the needs of cultural groups [19], a lack of cultural knowledge and awareness among healthcare practitioners, as well as insensitivity to cultural beliefs and practices, are implicated as key drivers of this inequality [24-26]. Cultural tailoring of DSMES programs to make them sensitive and responsive to health beliefs, practices, and linguistic needs of cultural groups has been shown to enhance improvements in important outcomes, including glycemic control (glycated hemoglobin [HbA_1c_]), knowledge, and QoL [26-28]. However, few have been evaluated in the United Kingdom [29]. Healthy Eating & Active Lifestyles for Diabetes (HEAL-D) is an evidence-based DSMES program, tailored to the cultural needs of Black African and Black Caribbean adults, which was co-designed with Black African and Black Caribbean people living with T2D, healthcare practitioners and commissioners, and community leaders [29-33]. Patient acceptability of HEAL-D has been demonstrated in a feasibility trial [34], but it is not known if the intervention is clinically and cost-effective.

As the first UK-based clinical trial to evaluate a culturally tailored DSMES program, the HEAL-D trial provides an opportunity to inform the future delivery of T2D care to Black African and Black Caribbean populations that have traditionally been underrepresented in clinical research and underserved by healthcare systems. By doing so, this trial can help to address several healthcare priorities, particularly relating to ethnic inequalities in healthcare outcomes [35-38]. If effective, the HEAL-D intervention could offer healthcare systems equitable DSMES services that meet the needs of some of its most vulnerable service users, ultimately reducing the health and economic burden of T2D. Furthermore, the outcomes of this trial could help inform and improve the implementation of healthcare programs for ethnic minority groups more broadly, helping to address health inequalities.

### Aims

The primary aim of this trial is to evaluate the effectiveness of the HEAL-D intervention, compared to standard DSMES programs, on glycemic control (assessed via HbA_1c_) at 12 months in Black African and Black Caribbean adults living with T2D. It is hypothesized that the HEAL-D intervention will improve glycemic control to a greater extent than standard DSMES programs at 12-month follow-up. Secondary aims include testing the effectiveness of HEAL-D, compared to standard DSMES programs, on cardiovascular risk factors; psychological well-being and QoL; T2D knowledge and self-efficacy; and diet and physical activity behaviors at 6, 12, and 24 months, as well as assessing cost-effectiveness. A mixed methods process evaluation aims to assess HEAL-D delivery, intervention fidelity and implementation, and the impact of multiple long-term conditions (MLTC) on recruitment and engagement with the HEAL-D intervention, and the impact of HEAL-D on MLTC.

## Methods

### Study Design

**Figure 1:**
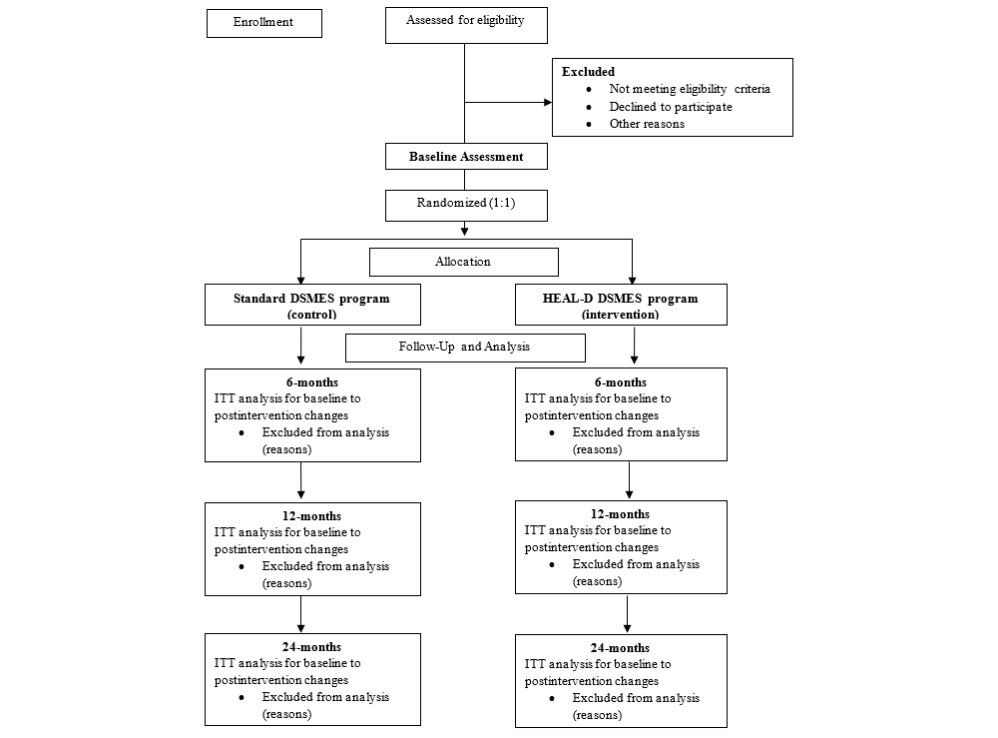
Trial CONSORT flow diagram. CONSORT: Consolidated Standards of Reporting Trials; DSMES: diabetes self-management education and support; HEAL-D: Healthy Eating and Active Lifestyles for Diabetes; ITT: intention-to-treat.

The trial is a 24-month, multicenter, pragmatic, open-label, 2-arm, parallel-group, individually randomized group treatment trial assessing the clinical and cost effectiveness of the HEAL-D program compared with standard DSMES programs in Black African and Black Caribbean adults living with T2D. A total of 300 Black African and Black Caribbean adults living with T2D will be recruited and randomly allocated to either the HEAL-D program (n=150) or a standard DSMES program (control, n=150). Outcome assessments will be conducted at baseline and 6, 12, and 24 months after randomization (Figure 1). Embedded within the trial are an internal feasibility assessment of recruitment, allocation and intervention engagement; a health economic analysis; a mixed methods process evaluation of intervention delivery, fidelity, implementation and acceptability; and study within a project (SWAP) to assess the impact of MLTC on the uptake of and engagement with HEAL-D, and the impact of the intervention on MLTC.

The trial is funded by the National Institute for Health Research (NIHR) Health Technology Assessment Program (NIHR151372) and is registered with ISRCTN (14344948), which adheres to the World Health Organization (WHO) Trial Registration Dataset. Health Research Authority and Research Ethics Council approval was received on April 22, 2024.

### Setting

The trial will be conducted in 3-5 centers in the United Kingdom, including London, West Midlands, and Greater Manchester, which have been selected as areas in the United Kingdom with significant representation of Black African and Black Caribbean adults. The trial will be centrally coordinated by the Diabetes Research Centre (University of Leicester, United Kingdom) and supported by infrastructure provided through the NIHR Research Delivery Network.

### Participants

A sample of 300 participants will be recruited. Inclusion criteria are: (1) adult (aged 18 years or older); (2) Black African or Black Caribbean ethnicity (self-declared and classified according to the Census system used within National Health Service (NHS) services: people of “Black African,” “Black Caribbean,” “Black British,” “Black Other,” and “mixed race” with either African or Caribbean ancestry); (3) T2D diagnosis (confirmed by medical history); (4) HbA_1c_≤100 mmol/mol (or fructosamine <450 µmol for individuals with sickle cell trait or disease); (5) Suitable for group-based training and participation in physical activity (suitability confirmed by a general practitioner or referring healthcare practitioner); (6) willing to undergo randomization; and (7) able to provide informed consent.

Exclusion criteria are (1) current pregnancy; (2) complex medical, lifestyle, or learning needs that require personalized advice or for which group-based training is unsuitable (eg, advanced chronic kidney disease and people with learning disabilities, confirmed by general practitioner or referring healthcare practitioner); (3) need for language translation services (spoken or written); (4) unable or unwilling to provided informed consent; and (5) current participation in competing clinical trial as determined by trial investigator.

The eligibility criteria have been selected pragmatically to align with UK clinical practice, whereby group-based DSMES is a recommended management option for most patients with T2D. However, patients with uncontrolled T2D (ie, high HbA_1c_) or specific or complex medical, educational, or lifestyle needs would not typically be referred to group-based DSMES programs, but would be managed in services that can provide more individualized advice or care. Similarly, patients needing language translation services would not typically be referred to group-based DSMES programs because of difficulties meeting their language needs.

### Recruitment and Screening

The research team will work with healthcare providers and grassroots or community organizations to identify and recruit participants. Several means of recruitment will be used: (1) screening of referrals to DSMES services from primary, intermediate or community care, and secondary care clinics; (2) primary care database searches; and (3) self-referral in response to study advertisements and community engagement activities.

Potential participants will be sent a participant information video via SMS text message or a written information leaflet via email or letter, providing a brief explanation of the purpose of the trial and participation requirements. Interested individuals will be invited to contact the research team or a community champion by phone, email, or web form to discuss the trial in detail and schedule a telephone eligibility screening, which will be conducted by the research team. Eligible participants, who confirm their intention to participate, will be invited to attend an in-person visit where written informed consent will be recorded prior to baseline data collection.

### Randomization and Blinding

Following the baseline assessment, each participant will be randomly allocated (1:1 ratio) to the intervention or control group using a centralized web-based randomization database (REDCap [Research Electronic Data Capture; Vanderbilt University]). Allocation will use randomly permuted blocks of variable block length and be stratified by center, accounting for the provision of different standard DSMES programs between centers, and baseline HbA_1c_ (48-52, 53-76, and 77-100 mmol/mol). The participants and researchers will not be blinded to group allocation.

### Intervention Group

HEAL-D is a culturally tailored DSMES program, consisting of 16 hours (eight 2-h sessions delivered over 6 mo) of group-based T2D self-management education and support, including participatory physical activity classes. The program is delivered by a diabetes specialist dietitian (no specified ethnicity), a community facilitator of Black African and Black Caribbean ethnicity, and exercise instructors (no specified ethnicity), using F2F or remote videoconferencing delivery modes; participants will choose their mode of attendance. HEAL-D aligns with quality standards [19], is underpinned by an evidence-based curriculum [39], and uses evidence-based behavior change techniques, informed by the Behavior Change Wheel and the Capability Opportunity Motivation-Behavior (COM-B) framework [40], to support adoption and long-term maintenance of the following diet and lifestyle goals [39]: (1) achieve 5%-10% weight loss or weight maintenance in those of healthy weight, (2) undertake ≥150 minutes per week of moderate to vigorous intensity aerobic physical activity plus 2 sessions per week of strength training, (3) balance carbohydrate intakes through portion control and promotion of low glycemic index and wholegrain sources, (4) limit saturated fat intake (<10% of energy intake) and replace with mono-unsaturated fats, (5) limit salt intake to no more than 6 g per day, and (6) consume at least 2 portions of oily fish per week.

Culturally tailored resources have been developed and are used to deliver the curriculum, including diet booklets, portion size guides, and participatory games focusing on cultural foods and dishes, and videos including health and motivational messages. Participants will be invited to bring a “significant other” to the program, but this is not compulsory. Sessions 1-7 will be delivered weekly or fortnightly, and Session 8 will be delivered 6 months after the start of the program. F2F delivery will be in community settings, such as faith-based venues and community centers, aiming for 8-12 patients in each group, and remote delivery will be via a videoconferencing platform, aiming for 6-8 patients in a group (remote group sizes will be smaller than F2F to facilitate the development of a supportive group dynamic whilst using a videoconferencing platform).

The HEAL-D intervention was developed in an earlier program of research funded by the NIHR; the intervention development process and findings and evaluation of patient acceptability have been published previously [30-32,34,41-43].

### Control Group

Control group participants will be referred to attend the standard NHS-commissioned DSMES course that is delivered in their local area and will be offered the choice of attending F2F or remote delivery, where both delivery methods are available. DSMES courses are a core NHS service with management guidelines recommending that all people with T2D attend a DSMES course [19], and referral is incentivized in primary care [44]. The content and structure of DSMES courses are guided by a quality framework, requiring courses to be group-based, delivering an evidence-based curriculum to support self-management skills, and be delivered by skilled, competent staff [19]. A range of courses are delivered in the NHS, typically providing 6-14 hours of group-based education and support; these courses are typically based on a standardized curriculum that does not include culturally tailored information or advice. HEAL-D has been developed to align with the quality standards for DSMES programs, but it is different from existing NHS courses in several ways. Principally, HEAL-D has been developed through a rigorous co-design process to reflect African and Caribbean cultural health beliefs and practices, providing tailored information, advice, and support through culturally sensitive resources. Furthermore, delivery is led by a combination of healthcare professionals (dietitians), culturally concordant “lay” facilitators, and exercise trainers, and the program includes participatory physical activity classes and practical cooking workshops.

In both trial arms, information on the factors that drove the choice of attendance mode will be collected. Following randomization, participants will attend their allocated DSMES program within 4 weeks, where possible, and the time from randomization to attendance will be recorded.

### Data Collection Procedures

As depicted in Figure 1, data will be collected prerandomization (baseline), 6 months, 12 months (primary endpoint), and 24 months post randomization. The primary outcome, secondary and exploratory outcomes, along with their associated timepoints of administration, are listed in Table 1. The measures will be collected during an in-person assessment of approximately 2 hours duration at an NHS research or primary care clinic facility. It is intended that all measures be completed on a single day, but they may occur across a maximum 14-day window. This might include completion of questionnaires via telephone or video call, or primary care or home visits for anthropometric or questionnaire measures. All staff will be trained in best practices for data collection and management by the trial manager, who also monitors data quality. Outcome assessors of the primary outcome (HbA_1c_) and other blood measurements will be blinded to treatment allocation; however, it is not possible to ensure blinding of assessors for the other outcome measures. Trial data will be recorded on a centralized web-based data collection platform (REDCap).

Multiple strategies will be implemented to maximize participant retention and completion of outcome assessments, including offering outcome assessments at different times of the day, remuneration for completion of outcome assessments (£50 [US $68] per assessment visit plus travel costs; there is no remuneration for participation in treatment sessions). Participants will be encouraged to complete outcome assessments, even if they did not attend treatment sessions or missed a previous outcome assessment. Participants will also receive brief study review contacts (via telephone or video calls) from the research team at three monthly intervals where assessment visits are not scheduled, to review ongoing willingness to continue.

**Table 1:** Study measures and assessment timepoints.

Outcome measure			Timepoint			
			Baseline	6 months	12 months	24 months
**Clinical variables**						
	Demographic information: age, sex, ethnicity, birthplace and generational status, employment status, education, marital status, socioeconomic status (index of multiple deprivation), all assessed by self-report		✓^a^			
	Medical history and medications: medical history (previous or current conditions), type 2 diabetes duration, and medication history, all assessed by self-report		✓			
**Primary outcome**						
	Glycemic control: HbA_1c_^b^ (mmol/mol); nonfasting blood sample		✓		✓	
**Secondary outcomes**						
	Glycemic control: HbA_1c_ (mmol/mol); nonfasting blood sample		✓	✓		✓
	Anthropometry and body composition					
		Body weight (kg); measured without shoes and removal of heavy clothing	✓	✓	✓	✓
		Body mass index (kg/m^2^); calculated from weight and height, measured without shoes and removal of heavy clothing	✓	✓	✓	✓
		Waist circumference (cm); measured at midpoint between lowest rib and iliac crest, over light clothing	✓	✓	✓	✓
		Body fat mass (kg); measured using bioelectrical impedance analysis	✓	✓	✓	✓
		Body lean mass (kg); measured using bioelectrical impedance analysis	✓	✓	✓	✓
	Cardiovascular risk markers					
		Total cholesterol (mol/L); nonfasting blood sample	✓	✓	✓	✓
		HDL^c^-cholesterol (mmol/L); nonfasting blood sample	✓	✓	✓	✓
		Systolic blood pressure (mm Hg); three seated measurements following 5 minutes rest, record the mean of the last two measures	✓	✓	✓	✓
		Diastolic blood pressure (mm Hg); three seated measurements following 5 minutes rest, record the mean of the last two measures	✓	✓	✓	✓
	Psychological well-being and quality of life					
		EQ-5D-5L quality of life questionnaire	✓	✓	✓	✓
		PAID-5^d^ questionnaire	✓	✓	✓	✓
		PHQ-9^e^	✓	✓	✓	✓
	Diabetes knowledge and self-efficacy					
		SDKI^f^	✓	✓	✓	✓
		DMSES-UK^g^ questionnaire	✓	✓	✓	✓
		PDDC^h^ questionnaire	✓	✓	✓	✓
	Lifestyle behaviors					
		s-IPAQ^i^	✓	✓	✓	✓
		7 to 10-day arm or wrist-worn accelerometry; sleep, inactivity, step count, total physical activity, and moderate to vigorous physical activity	✓		✓	
		DQQ^j^	✓	✓	✓	✓
**Tertiary or exploratory outcomes**						
	Blood lipids					
		LDL^k^-cholesterol (mmol/L); nonfasting blood sample	✓	✓	✓	✓
		Triglyceride (mmol/L); nonfasting blood sample	✓	✓	✓	✓
	Body composition: body fat (kg and %); measured using bioelectrical impedance analysis		✓	✓	✓	✓
	Lifestyle behaviors: self-reported sleep duration and chronotype		✓	✓	✓	✓
	Medication use					
		Glucose-lowering therapies use, assessed via self-reported medication history	✓	✓	✓	✓
		Blood pressure-lowering therapies use, assessed via self-reported medication history	✓	✓	✓	✓
	Multimorbidity status					
		Diagnoses or remission or changes in severity of medical conditions, assessed via self-reported medical history	✓	✓	✓	✓
		MTBQ^l^	✓	✓	✓	✓
**Health economics measures**						
	Health service use: AD-SUS^m^ adapted; self-report log of health service use		✓	✓	✓	✓
**Process measures**						
	Intervention acceptability: quantitative evaluation questionnaire				✓	
	Intervention delivery, implementation, and fidelity: session observations and checklists Qualitative interview or workshop with participants and intervention delivery staff				✓	

^a^Outcome is assessed at this timepoint only.

^b^HbA_1c_: hemoglobin A_1c_.

^c^HDL: high-density lipoprotein.

^d^PAID-5: 5-point Problem Areas In Diabetes.

^e^PHQ-9: Patient Health Questionnaire-9.

^f^SKDI: Short Diabetes Knowledge Instrument.

^g^DMSES-UK: Diabetes Management Self-Efficacy Scale-UK.

^h^PDDC: Perceived Diabetes and Dietary Competence.

^i^s-IPAQ: Short International Physical Activity Questionnaire.

^j^DQQ: Diet Quality Questionnaire.

^k^LDL: low-density lipoprotein.

^l^MTBQ: Multimorbidity Treatment Burden Questionnaire.

^m^AD-SUS: Adult-Service Use Schedule.

### Primary Outcome

The primary outcome upon which the trial is powered is the difference between groups in the change in HbA_1c_ from baseline to 12 months. HbA_1c_ was chosen for several reasons: it is the principal clinical measure of diabetes status and glycemic control, and a valuable surrogate measure of holistic engagement with diabetes management and self-care; reduction of HbA_1c_ is associated with reduced risk of micro and macrovascular complications, and in some cases, all-cause mortality [45]; it is also a prominent component of the Core Outcome Measures in Effectiveness Trials initiative core outcomes set for T2D [46]. A 12-month primary endpoint has been chosen to examine the effectiveness of the HEAL-D intervention, as this is a duration long enough to observe a clinically important difference in HbA_1c_.

### Secondary and Exploratory Outcomes

At 6 and 24 months, HbA_1c_ will be measured as a secondary outcome to allow exploration of the time-course of any observed changes and impact of HEAL-D over a longer period. Other secondary outcomes (Table 1), measured at 6, 12, and 24 months, are grouped into holistic health domains and include cardiovascular risk factors, psychological well-being and self-management support, lifestyle behaviors, and health economics.

Tertiary outcomes are low-density lipoprotein-cholesterol, triglycerides, body fat percentage, changes to glucose-lowering and antihypertensive therapies (including addition, removal, or dose adjustment), and changes in MLTC status (including additional diagnoses, remission, or changes in severity).

### Measurements

At baseline and all follow-up visits, unless specified otherwise, the following measurements will be conducted.

#### HbA_1c_ and Blood Lipids

A 5 mL venous blood sample (nonfasting) will be taken via venepuncture according to local standardized operating procedures for measurement of HbA_1c_ and full lipid profile by the pathology department at the corresponding clinical site. Biological samples taken for the study will be destroyed once analyzed in accordance with the Human Tissue Act 2004.

#### Blood Pressure

Brachial arterial blood pressure will be measured in the seated position using an automated sphygmomanometer after participants have been resting for ~5 minutes. Three blood pressure measurements will be obtained, and the average of the last two measurements will be used.

#### Anthropometry

Body weight will be measured using digital scales, with the patient wearing light clothing (without shoes), to the nearest 0.1 kg. Height will be measured to the nearest 0.5 cm, using a stadiometer, without shoes. Waist circumference will be measured using a flexible tape, with the patient wearing only light clothing, using the WHO methodology, which defines the “waist” as the midpoint between the lowest rib and the iliac crest. The mean of three waist circumference measurements will be recorded. Body composition will be measured using Tanita DC-430-MA P bioelectrical impedance scales; body fat (%) and lean mass (kg) will be recorded.

#### Physical Activity

Physical activity will be measured objectively, using a wrist-worn accelerometer over 7-10 days, measuring sleep, inactivity, step count, total physical activity, and moderate to vigorous physical activity. Accelerometer use will occur at baseline and the 12-month visits only. Self-reported physical activity will be recorded using the short International Physical Activity Questionnaire, which consists of seven questions about the amount of time spent in different levels of physical activity (vigorous, moderate, and low intensity), categorizing overall physical activity levels as low, moderate, or high [47].

#### Patient Reported Outcome Measures

Several patient-reported outcome measures, including diabetes-specific measures, will be collected:

- QoL will be assessed using EQ-5D-5L [48] and used in the cost-effectiveness evaluation.
- Diabetes-related distress will be assessed using the 5-point Problem Areas in Diabetes (PAID-5) questionnaire [49]; PAID-5 is widely used in diabetes trials as an indicator of diabetes-specific QoL.
- Depressive symptoms will be assessed using the Patient Health Questionnaire-9 (PHQ-9) [50]; PHQ-9 is used widely, including in the NHS, to assess symptoms of depression.
- Diabetes knowledge will be assessed using the Short Diabetes Knowledge Instrument (SDKI) [51].
- Diabetes self-efficacy will be assessed using the Diabetes Management Self-Efficacy Scale [52].
- Diabetes dietary competence will be assessed using the Perceived Diabetes and Dietary Competence (PDDC) [53].
- Multimorbidity treatment burden will be assessed using the Multimorbidity Treatment Burden Questionnaire [54].
- The Diet Quality Questionnaire will be used to assess dietary adequacy, providing a measure of diet diversity and protection against noncommunicable diseases [55].
- Health service resource utilization will be assessed using an adapted Adult Service Use Schedule [56] and will be used to inform the health economic evaluation.
- Self-reported sleep duration and chronotype will each be assessed using an individual question adapted from the Morningness-Eveningness Questionnaire [57].

The EQ-5D-5L, PAID-5, PHQ-9, SDKI, DSMES-UK, and PDDC have evidenced reliability and validity for use in people with T2D [48-53]; none of the questionnaires have been validated specifically in populations of African and Caribbean heritage, although the SDKI and PDDC have been used in studies with African-American populations. The questionnaires will be interviewer-led or self-completed, depending on participant preference or literacy.

#### Internal Feasibility Assessment

A feasibility assessment will be conducted in the first 6 months of the trial to establish the feasibility of completing the trial within desired timelines, focused on (1) identification of eligible participants, (2) consent and randomization of eligible participants, and (3) engagement with treatment allocation of randomized participants. Recruitment and intervention engagement data will be reviewed by the Trial Steering Committee (TSC) with predefined “stop/go” criteria to determine progression to full trial.

#### Health Economic Evaluation

The primary aim of the health economic evaluation will be to assess the within-trial incremental cost-effectiveness of both remote and F2F versions of HEAL-D compared to standard DSMES programs. This will be undertaken through a cost-utility analysis with participant outcomes measured in quality-adjusted life years (QALYs) gained. Secondary aims include: (1) whether within-trial cost-effectiveness conclusions are affected by a consideration of long-term cost and QALY outcomes extrapolated from observed clinical end points within the clinical trial, and (2) whether the program is likely to be cost-effective when delivered at scale in routine practice, accounting for costs of implementation, such as facilitator training, and expected population reach or engagement levels.

Inferences regarding the cost-effectiveness of HEAL-D will be made with reference to the incremental cost-effectiveness ratio, applying varying cost-effectiveness thresholds (inclusive of those adopted by The National Institute for Health and Care Excellence) for identifying whether new health programs offer the NHS sufficient value for money [58]. The resource and cost implications of HEAL-D will be evaluated from an NHS or Personal Social Services perspective. All analyses will be undertaken probabilistically to reflect uncertainty in key economic and clinical parameters of relevance.

The short-term costs and benefits of HEAL-D will be quantified using data collected over the trial period, including resource inputs allocated to program delivery and implementation (eg, training activity), wider service utilization among trial participants over follow-up (using the self-report Adult Service Use Schedule), and short-term health-related QoL outcomes (based on the EQ-5D-5L instrument). For secondary analysis, long-term resource and QALY impacts will be estimated using the UKPDS Outcomes model [59]. The UKPDS is a microsimulation modeling tool that can be applied to make extrapolations regarding the incidence of complications (micro and macrovascular) and associated costs, QoL, and survival trajectories.

In further secondary analysis, we will draw on data and evidence from the main evaluation of program cost-effectiveness to assess whether it would be cost-effective to deliver alternative versions of HEAL-D at scale within routine service settings within a defined locality and population (South London). We will use existing frameworks and toolkits [60,61] to estimate the potential costs of implementation at scale and will evaluate the cost-effectiveness of scale-up, allowing for implementation costs and population engagement or reach.

#### Mixed Methods Process Evaluation

An embedded mixed methods process evaluation, combining relevant data gathered within the trial (questionnaires, monitoring data), qualitative interview data, logbook entries, workshops, and observations, will provide a formative evaluation of the intervention’s implementation, mechanisms of action, and identification of the contextual factors that influence its implementation and adoption. Implementation will encompass: fidelity (whether training and the intervention sessions were delivered as intended, whether healthcare professionals delivered culturally sensitive behavior change support, and any observed barriers to this), intervention dose (ie, attendance and completion rates), and reach (whether the intended audience encounters the intervention, and how). Mechanisms of action will encompass: satisfaction (with the program), participant and facilitator experience of the intervention, and effective and less effective components of the intervention in engaging participants and producing desired results (eg, increasing physical activity) and potential reasons. The identification of contextual factors that influence the implementation and adoption of HEAL-D includes implications for workforce capacity and for intervention integration within existing care.

Participants who are randomized to the HEAL-D intervention arm and staff involved in HEAL-D delivery will be eligible to participate in the process evaluation. We aim to recruit a total of 48 participants (16 per center) based on sampling for diversity in relation to high versus low intervention engagement, F2F versus remote attendance, and MLTC versus non-MLTC status. Maximum variation in sampling of participants will be guided by age, gender, employment status, and ethnicity (Black African or Black Caribbean). The characteristics of the sample will be continually reviewed to achieve balanced representation, inviting consecutive participants until the target sample and data saturation have been achieved. We aim to recruit up to 27 staff (9 per center), including delivery staff and trainers. Staff must meet the following criteria to participate: clinical and nonclinical staff working at or in partnership with centers participating in the HEAL-D trial, aged 18 years or older, and able to give written informed consent.

Data for the process evaluation will be collected via several means. First, both the training of HEAL-D facilitators and the delivery of HEAL-D sessions by facilitators will be observed. A self-report questionnaire, completed at the 12-month visit, will be used to assess the acceptability of trial procedures by participants. Participants will also be invited to undertake a semistructured interview within 1 month after the 12-month visit. These interviews will be conducted by telephone and provide an open and flexible method for exploring individual experiences in-depth. Participants and staff will also be invited to attend a F2F workshop to explore HEAL-D implementation.

#### SWAP

The SWAP aims to explore uptake of and engagement with HEAL-D in adults with T2D who also have MLTC, and has the following objectives: (1) to assess the prevalence of MLTC in adults of Black African or Black Caribbean ethnicity participating in the HEAL-D trial and identify and describe common MLTC clusters; (2) to assess the impact of MLTC on uptake, engagement with, and completion of HEAL-D; (3) to assess the impact of the HEAL-D intervention on associated MLTC; and (4) to codevelop dissemination outputs in a workshop with a patient and public involvement and engagement (PPIE) group and other relevant stakeholders, ensuring strong representation of those with MLTC. The SWAP will be conducted in two phases: quantitative data collection, to address objective 1, and qualitative data collection to address objectives 2-4. All participants will be eligible for the SWAP and have quantitative data collected (eg, prevalence and details of MTLC); only participants randomized to the intervention arm will be eligible to participate in the qualitative data phase (ie, semistructured interviews and workshops).

All study participants will complete a questionnaire to elicit background information on the long-term conditions they experience (with MLTC defined as per Guthrie et al [62]). The extent to which these conditions affect their day-to-day living will be assessed using the Multimorbidity Treatment Burden Questionnaire. This questionnaire will be analyzed together with participant demographic data and baseline exercise capacity, QoL, and psychological assessments. A subsample of participants who are randomized to the HEAL-D arm will undertake an interview focusing on: experience of T2D, experience of MLTC, uptake of and engagement with the HEAL-D intervention (both F2F and remote attendance), trial experience, trial completion, and interaction between MLTC and intervention. These interviews will be embedded in the process evaluation and account for 16/48 of the participant interviews. Qualitative data will also be obtained within the three main process evaluation workshops, where additional needs of people with MLTC, the burden of MLTC, and barriers and facilitators to acceptance of HEAL-D will be discussed. For the SWAP, there will also be two additional workshops to deliberate on whether the intervention could be optimized for MLTC and what lessons can be shared with other stakeholders going forward.

### Sample Size Calculation

The trial is based on a minimal clinically important difference in HbA_1c_ of 5 mmol/mol, leading to significant risk reductions for T2D complications [19]. Power was calculated at 90%, with a 5% 2-sided significance level, to detect a standardized effect size of 0.45 (difference in HbA_1c_ of 5 and SD of 11 mmol/mol, determined from the feasibility trial [34] and unpublished primary care data from South London). To allow for correlation of outcomes among group attendees, the sample size, assuming no correlation (103 per arm), is inflated by a design effect (1.09) and then rounded up to ensure divisibility by group size. The F2F intervention is to be delivered in groups of up to 12, while the remote intervention will be delivered in groups of up to 8; for the calculations, an average group size of 10 is assumed. Given the objective outcome, the short duration of treatment, patterns observed in the intracluster correlation coefficient for cluster randomized trials suggest an intracluster correlation coefficient of 0.01 [63]. In the previous feasibility trial, loss to follow-up was 7% at 6 months. Given the pragmatic design, whereby participants are given free choice as to their mode of attendance (F2F or remote) in both the intervention and comparator arms, we do not expect substantial differences in retention between arms. However, with a longer primary outcome follow-up of 12 months, loss to follow-up is estimated to be higher at 15%. For the intervention arm, this is accounted for by increasing the number of clusters. Therefore, we will recruit 150 participants in the control arm and 150 participants in the intervention arm, each across 15 groups of average size 10.

### Statistical Analysis

Primary analysis of change in HbA_1c_ at 12 months will be conducted using a mixed effects model with a random effect for the group attended; individuals in the control arm will be treated as groups of size 1 [64]. Treatment arm, center, and baseline HbA_1c_ will be included as fixed effects. The primary analysis will be repeated to include an interaction term between treatment and mode of delivery (F2F or remote) to explore any differential treatment effect among groups. The presence of an interaction will be tested using a likelihood ratio test. The prespecified subgroup analyses of the primary outcome will assess whether the effectiveness of the intervention is dependent on baseline HbA_1c_ or center. This will be assessed by adding interaction terms between group allocation and the potential effect modifiers to the linear regression, one at a time. The analysis exploring the change in treatment effect over time for the secondary outcomes will follow the methodology of the primary analysis, with an additional random effect for the individual to account for within-individual correlation over time, a fixed effect for time, and an interaction between treatment and time effect. Mediation analysis will be conducted using multilevel structural equation modeling [65]. To account for missing data, multiple imputation will be undertaken, provided we have strong predictors of missingness and an appropriate imputation model. Diagnostic checks will be performed to assess this. The missing data mechanism will be assumed to be missing at random. Individual analyses on each imputed dataset will be combined using the Rubin rules. Sensitivity analyses will be conducted to assess the robustness of the primary conclusions to the imputation strategy used.

For data collected during the mixed methods process evaluation, deductive and inductive thematic analysis of interviews, workshops, logs, and observations, using NVivo (QSR International) Framework, will be followed by triangulation of qualitative and quantitative data using a meta-matrix [66]. We will examine divergence and similarity across study groups (eg, remote vs F2F, by center), and within the triangulated data to develop a comprehensive understanding of intervention delivery and fidelity, recruitment and engagement, and mechanisms of impact. Thematic analysis [67] will be undertaken with the interview transcripts, informed by the Theoretical Domains Framework and the COM-B framework, toward identifying barriers and facilitators for engagement and maintenance of behavior change [63]. For the thematic analysis, using NVivo software, two researchers will independently double-code 10% of randomly selected transcripts to develop a coding framework. Further transcripts will be double-coded one by one until interrater reliability is such that the framework is agreed upon. Remaining transcripts will then be single-coded (ie, by one of the two researchers). Themes will be developed through discussion with the wider team. To indicate the frequency with which themes occur, we will use “all” (100%), “almost all” (>85%), “most” (>75%), “the majority” (>50%), “some” (>10%), and “a few” (≥10%). We will use the Consolidated Criteria for Reporting Qualitative Research tool [68]. The health economic evaluation analysis has been described in the relevant section.

### Safety Reporting

As the trial is not a clinical trial of an investigational medicinal product, monitoring of pharmacovigilance is not relevant. Adverse events will be recorded and discussed periodically with the TSC as required. Any safety concerns that arise as a result will be reported to the sponsor as soon as possible. Serious adverse events (SAEs) will be reported to the sponsor within 24 hours of becoming aware of the event. The principal investigator or another delegated physician (as agreed by the sponsor) will be responsible for the review and sign-off of the SAE and the assessment of causality (ie, whether an event is related to a study procedure or intervention). Events will be followed up on until the event has been resolved or a final outcome has been reached. In addition to the expedited reporting above, the chief investigator (CI) shall submit, once a year throughout the study or on request, an Annual Report to the Ethics Committee, which lists all SAEs.

### Posttrial Care

Provisions will be put in place for posttrial access for all participants who still need an intervention identified as individually beneficial in the trial. This includes referral to local providers of physical activity services and psychosocial support (ie, primary care services).

### Ethical Considerations

The trial has been approved by the Health Research Authority (IRAS ID: 326064) and East Midlands—Leicester South Research Ethics Committee (REC reference: 24/EM/0079). The trial will be conducted in full conformity with the current revision of the Declaration of Helsinki (last amended October 2000, with additional footnotes added 2002 and 2004), the UK Policy Framework for Health and Social Care Research (2017), and International Council for Harmonization of Technical Requirements for Pharmaceuticals for Human Use Good Clinical Practice relevant regulations. Potential participants who express an interest in participating in the trial will be provided with an information video or leaflet providing full details of the trial and what is entailed. Participants will be given an opportunity to discuss participation with a trial coordinator. Those who decide to participate will be required to provide written informed consent prior to the collection of research data; consent will be collected by trained research staff at the baseline assessment visit. Participant data will be pseudonymized and treated in confidence and in compliance with the International Council for Harmonization of Technical Requirements for Pharmaceuticals for Human Use Good Clinical Practice, the UK Policy Framework for Health and Social Care, and the UK General Data Protection Regulation. All investigators and trial site staff will comply with the requirements of the Data Protection Act or General Data Protection Regulation with regard to the collection, storage, processing, and disclosure of personal information and will uphold the Act’s core principles. The CI will have access to the trial documentation and will be the data custodian. Participants will receive £50 (US $68) for each of the assessment visits they attend in compensation for their time commitment and will have their travel costs reimbursed. Individuals who participate in an interview for the process evaluation will receive £20 (US $27) to compensate for their time. Participants will not receive financial reimbursement for attending their allocated treatment.

### Study Oversight and Monitoring

This trial is sponsored by The University of Leicester, United Kingdom (reference: 0928). The University of Leicester operates a risk-based monitoring and audit program, to which this study will be subject. The Pragmatic Clinical Trials Unit, Queen Mary University of London, United Kingdom, will provide support regarding data management, statistical analysis, and quality assurance. A TSC, consisting of an independent statistician, health economist, person of Black African and Black Caribbean ethnicity with lived experience of T2D, and other independent subject specialists, has been formed and will offer independent strategic oversight throughout the trial. In line with the European Medicines Agency Guideline on data monitoring committees [69], the sponsor determined that a Data Monitoring and Ethics Committee is not required for the trial.

### Protocol Amendments

Amendments will be submitted to the sponsor in the first instance for review and approval. The CI, in agreement with the sponsor, will then submit information to the appropriate body (HRA or REC) for approval. Any amendments will also be approved by the trial funder and communicated to the trial registry.

## Results

The trial, funded by the NIHR, will run for 48 months in total (August 1, 2023, to July 31, 2027). Site “green light” was received on August 15, 2024, for London; November 29, 2024, for Manchester; and January 31, 2025, in the West Midlands. Recruitment commenced in August 2024 and is due to run over 11 months. As of March 26, 2025, a total of 76 participants have consented, and 72 participants have been randomized. The “last patient, last visit” is expected in June 2027; primary data analysis is expected to begin in July 2027. Final results are anticipated to be available in September 2027, and publication is expected by the end of 2027.

## Discussion

### Overview

The HEAL-D trial will evaluate if a culturally tailored DSMES program, provided in person or remotely, via videoconferencing, is clinically and cost-effective, in comparison with standard DSMES programs, at improving glycemic control (HbA_1c_) at 12-month follow-up in adults of Black African and Black Caribbean ethnicity living with T2D. It is hypothesized that the provision of a DSMES program that is sensitive to the cultural health beliefs and practices of adults of African and Caribbean heritage will promote clinically meaningful improvements in T2D self-management.

### Principal Findings

Principally, the trial is designed to evaluate the effectiveness of the HEAL-D intervention, compared to standard DSMES programs, on glycemic control at 12 months. In addition, a range of important clinical and patient-reported outcome measures, including cardiovascular risk factors, psychological well-being and QoL, T2D knowledge and self-efficacy, and diet and physical activity behaviors, will be collected at 6, 12, and 24 months. Importantly, the 24-month endpoint will enable assessment of the longer-term impact of the intervention on the clinical and patient-reported outcomes.

### Comparison to Prior Work

To date, in the United Kingdom, there have been no trial evaluations of culturally tailored DSMES programs for T2D self-management in adults of Black African and Black Caribbean ethnicity [29,70]. However, previous trials in the United States have demonstrated significant reductions in HbA_1c_, as well as improvements in T2D knowledge, which were associated with HbA_1c_ improvements [29].

### Strengths and Limitations

The HEAL-D trial builds on a large program of co-design research, which engaged a wide range of stakeholders (people of Black African and Black Caribbean ethnicity living with T2D, healthcare practitioners and commissioners, and community leaders) in the development of the intervention to ensure its relevance to both patients and healthcare service providers [29-33]. Furthermore, the acceptability of the HEAL-D intervention has been rigorously evaluated, and the feasibility of conducting a randomized controlled trial was assessed in earlier work [34,41-43,71,72]; the design of this trial has been informed by those evaluations, reflecting the main findings. People with lived experience of T2D continue to inform the delivery of the trial, as well as future dissemination, through involvement in the trial PPIE stakeholder group.

### Future Directions

A key concern in clinical practice is the accessibility of educational programs for the population in question. The development of both F2F and remote delivery formats hopefully maximizes the potential for pragmatic use in healthcare, whilst the availability of digital delivery may be particularly beneficial for ethnic minority groups [23]. For example, remote programs can reach those who are unable to attend F2F sessions [73] and can bring together individuals from areas with low population densities of a given ethnic group [23]. Digital interventions may also be more cost-effective, as spotlighted during the COVID-19 pandemic [74]. Furthermore, ongoing PPIE during the development of the HEAL-D intervention has enabled the production of a program and related materials that patients have found both engaging and accessible, as described previously [30-32]. By incorporating assessments of effectiveness, cost-effectiveness, a thorough process evaluation, and SWAP into the trial design, it is ensured that the needs of all stakeholders continue to be accounted for, thus promoting the feasibility of implementing the HEAL-D program within healthcare services.

### Dissemination Policy

The trial results will be disseminated via diverse and targeted means. All participants will receive a written plain-language summary of the results of the trial. A PPIE community engagement plan will be developed with our PPIE group, with plans for engagement and dissemination activities using a combination of platforms such as social media or website, radio, and written information via newspapers relevant to Black African and Black Caribbean communities, to ensure communication about the study reaches them. Ongoing review and revision of our dissemination strategy will be undertaken throughout the program, in partnership with PPIE partners, to maximize impact and ensure relevance and accessibility to a wide range of groups.

The trial results will be published in open-access medical journals and at conferences, with the intention of reaching a global audience. The outcome of this multicenter randomized controlled trial has the capacity to change practice and may be implemented in other English-speaking countries with minimal adaptation. It is anticipated that the findings of this study will be incorporated into national and international guidelines. Authorship will be determined in line with the International Committee of Medical Journal Editors guidelines.

## Appendix

Multimedia Appendix 1 CONSORT EHEALTH checklist.

Multimedia Appendix 2 Funder peer review reports.

## Abbreviations

CI: chief investigator
DSMES: diabetes self-management education and support
F2F: face-to-face
HbA_1c_: glycated hemoglobin
HEAL-D: Healthy Eating and Active Lifestyles for Diabetes
MLTC: multiple long-term conditions
NHS: National Health Service
NIHR: National Institute for Health and Care Research
PAID-5: 5-point Problem Areas in Diabetes
PDDC: Perceived Diabetes and Dietary Competence
PHQ-9: Patient Health Questionnaire-9
PPIE: patient and public involvement and engagement
QALY: quality-adjusted life year
QoL: quality of life
REDCap: Research Electronic Data Capture
SAE: serious adverse event
SDKI: Short Diabetes Knowledge Instrument
SWAP: study within a project
T2D: type 2 diabetes
TSC: Trial Steering Committee
WHO: World Health Organization

## Data Availability

The data that support the findings of this trial will be available from the corresponding author upon reasonable request.
