# Supplementary material for: Culturally Tailored Diabetes Self-Management Education and Support Programs in Black African and Caribbean Adults With Type 2 Diabetes (HEAL-D): Protocol for a Multicenter, Pragmatic Randomized Controlled Trial
Source: JMIR Res Protoc. 2025 Sep 30;14:e71861. doi: 10.2196/71861 (PMC12521847; doi:10.2196/71861)
Supplement: Multimedia Appendix 1 [file resprot_v14i1e71861_app1.pdf]

CONSORT-EHEALTH (V 1.6.1) - Submission/Publication Form

The CONSORT-EHEALTH checklist is intended for authors of randomized trials evaluating web-based and Internet-based applications/interventions, including mobile interventions, electronic games (incl multiplayer games), social media, certain telehealth applications, and other interactive and/or networked electronic applications. Some of the items (e.g. all subitems under item 5 - description of the intervention) may also be applicable for other study designs.

The goal of the CONSORT EHEALTH checklist and guideline is to be
a) a guide for reporting for authors of RCTs,
b) to form a basis for appraisal of an ehealth trial (in terms of validity)

CONSORT-EHEALTH items/subitems are MANDATORY reporting items for studies published in the Journal of Medical Internet Research and other journals / scientific societies endorsing the checklist.

Items numbered 1., 2., 3., 4a., 4b etc are original CONSORT or CONSORT-NPT (non-pharmacologic treatment) items.
Items with Roman numerals (i., ii, iii, iv etc.) are CONSORT-EHEALTH extensions/clarifications.

As the CONSORT-EHEALTH checklist is still considered in a formative stage, we would ask that you also RATE ON A SCALE OF 1-5 how important/useful you feel each item is FOR THE PURPOSE OF THE CHECKLIST and reporting guideline (optional).

Mandatory reporting items are marked with a red *.
In the textboxes, either copy & paste the relevant sections from your manuscript into this form - please include any quotes from your manuscript in QUOTATION MARKS,
or answer directly by providing additional information not in the manuscript, or elaborating on why the item was not relevant for this study.

YOUR ANSWERS WILL BE PUBLISHED AS A SUPPLEMENTARY FILE TO YOUR PUBLICATION IN JMIR AND ARE CONSIDERED PART OF YOUR PUBLICATION (IF ACCEPTED).
Please fill in these questions diligently. Information will not be copyedited, so please use proper spelling and grammar, use correct capitalization, and avoid abbreviations.

DO NOT FORGET TO SAVE AS PDF _AND_ CLICK THE SUBMIT BUTTON SO YOUR ANSWERS ARE IN OUR DATABASE !!!

Citation Suggestion (if you append the pdf as Appendix we suggest to cite this paper in the caption):
Eysenbach G, CONSORT-EHEALTH Group
CONSORT-EHEALTH: Improving and Standardizing Evaluation Reports of Web-based and Mobile Health Interventions
J Med Internet Res 2011;13(4):e126

URL: http://www.jmir.org/2011/4/e126/
doi: 10.2196/jmir.1923
PMID: 22209829

Sign in to Google to save your progress. Learn more

* Indicates required question

Your name *

First Last

Louise Goff

Primary Affiliation (short), City, Country *

University of Toronto, Toronto, Canada

University of Leicester, Leicester, UK

Your e-mail address *

abc@gmail.com

louise.goff@leicester.ac.uk

Title of your manuscript *

Provide the (draft) title of your manuscript.

Healthy Eating & Active Lifestyles for Diabetes (HEAL-D): protocol for a multicentre, pragmatic randomised controlled trial of culturally tailored versus standard diabetes self-management education and support programmes in black African and black Caribbean adults with type 2 diabetes

Name of your App/Software/Intervention *

If there is a short and a long/alternate name, write the short name first and add the long name in brackets.

HEAL-D (Healthy Eating and Active Lifestyles f

Evaluated Version (if any)

e.g. "V1", "Release 2017-03-01", "Version 2.0.27913"

Not applicable

Language(s) *

What language is the intervention/app in? If multiple languages are available, separate by comma (e.g. "English, French")

English

URL of your Intervention Website or App

e.g. a direct link to the mobile app on app in appstore (itunes, Google Play), or URL of the website. If the intervention is a DVD or hardware, you can also link to an Amazon page.

https://heal-d.org/

URL of an image/screenshot (optional)

Your answer

Accessibility *

Can an enduser access the intervention presently?

- ○ access is free and open
- ◉ access only for special usergroups, not open
- ○ access is open to everyone, but requires payment/subscription/in-app purchases
- ○ app/intervention no longer accessible
- ○ Other:

Primary Medical Indication/Disease/Condition *

e.g. "Stress", "Diabetes", or define the target group in brackets after the condition, e.g. "Autism (Parents of children with)", "Alzheimers (Informal Caregivers of)"

Type 2 diabetes

Primary Outcomes measured in trial *

comma-separated list of primary outcomes reported in the trial

HbA1c

Secondary/other outcomes

Are there any other outcomes the intervention is expected to affect?

Cholesterol, weight, BMI, waist circumference, blood pressure, body fat percentage, physical activity level, dietary intake, quality of life, diabetes distress, depressive symptoms, diabetes knowledge, diabetes self-efficacy.

Recommended "Dose" *

What do the instructions for users say on how often the app should be used?

- ◯ Approximately Daily
- ◯ Approximately Weekly
- ◯ Approximately Monthly
- ◯ Approximately Yearly
- ◯ "as needed"
- ◉ Other: 16 hours, delivered as 8 sessions of 2-hours duration, over a 6 month

Approx. Percentage of Users (starters) still using the app as recommended after 3 months *

- ◉ unknown / not evaluated
- ◯ 0-10%
- ◯ 11-20%
- ◯ 21-30%
- ◯ 31-40%
- ◯ 41-50%
- ◯ 51-60%
- ◯ 61-70%
- ◯ 71%-80%
- ◯ 81-90%
- ◯ 91-100%
- ◯ Other:

Overall, was the app/intervention effective? *

- ○ yes: all primary outcomes were significantly better in intervention group vs control
- ○ partly: SOME primary outcomes were significantly better in intervention group vs control
- ○ no statistically significant difference between control and intervention
- ○ potentially harmful: control was significantly better than intervention in one or more outcomes
- ◉ inconclusive: more research is needed
- ○ Other:

Article Preparation Status/Stage *

At which stage in your article preparation are you currently (at the time you fill in this form)

- ○ not submitted yet - in early draft status
- ○ not submitted yet - in late draft status, just before submission
- ○ submitted to a journal but not reviewed yet
- ◉ submitted to a journal and after receiving initial reviewer comments
- ○ submitted to a journal and accepted, but not published yet
- ○ published
- ○ Other:

Journal *

If you already know where you will submit this paper (or if it is already submitted), please provide the journal name (if it is not JMIR, provide the journal name under "other")

- ○ not submitted yet / unclear where I will submit this
- ○ Journal of Medical Internet Research (JMIR)
- ○ JMIR mHealth and UHealth
- ○ JMIR Serious Games
- ○ JMIR Mental Health
- ○ JMIR Public Health
- ○ JMIR Formative Research
- ○ Other JMIR sister journal
- ◉ Other: JMIR Research Protocols

Is this a full powered effectiveness trial or a pilot/feasibility trial? *

- ○ Pilot/feasibility
- ◉ Fully powered

Manuscript tracking number *

If this is a JMIR submission, please provide the manuscript tracking number under "other" (The ms tracking number can be found in the submission acknowledgement email, or when you login as author in JMIR. If the paper is already published in JMIR, then the ms tracking number is the four-digit number at the end of the DOI, to be found at the bottom of each published article in JMIR)

- ○ no ms number (yet) / not (yet) submitted to / published in JMIR
- ◉ Other: 71861

TITLE AND ABSTRACT

1a) TITLE: Identification as a randomized trial in the title

1a) Does your paper address CONSORT item 1a? *

I.e does the title contain the phrase "Randomized Controlled Trial"? (if not, explain the reason under "other")

- (●) yes
- ( ) Other:

1a-i) Identify the mode of delivery in the title

Identify the mode of delivery. Preferably use “web-based” and/or “mobile” and/or “electronic game” in the title. Avoid ambiguous terms like “online”, “virtual”, “interactive”. Use “Internet-based” only if Intervention includes non-web-based Internet components (e.g. email), use “computer-based” or “electronic” only if offline products are used. Use “virtual” only in the context of “virtual reality” (3-D worlds). Use “online” only in the context of “online support groups”. Complement or substitute product names with broader terms for the class of products (such as “mobile” or “smart phone” instead of “iphone”), especially if the application runs on different platforms.

	1	2	3	4	5	
subitem not at all important	(●)	( )	( )	( )	( )	essential

Clear selection

Does your paper address subitem 1a-i? *

Copy and paste relevant sections from manuscript title (include quotes in quotation marks "like this" to indicate direct quotes from your manuscript), or elaborate on this item by providing additional information not in the ms, or briefly explain why the item is not applicable/relevant for your study

"Healthy Eating & Active Lifestyles for Diabetes (HEAL-D) is a co-designed culturally tailored DSMES programme for adults of black African and black Caribbean ethnicity. HEAL-D provides group-based education, behaviour change support and participatory physical activity sessions, delivered either face-to-face (F2F) or via online video conferencing."

"The programme is delivered by a diabetes specialist dietitian (no specified ethnicity), a community facilitator of black African and black Caribbean ethnicity, and exercise instructors (no specified ethnicity), using F2F or online video conferencing delivery modes"

1a-ii) Non-web-based components or important co-interventions in title

Mention non-web-based components or important co-interventions in title, if any (e.g., “with telephone support”).

	1	2	3	4	5	
subitem not at all important	(●)	( )	( )	( )	( )	essential

Clear selection

Does your paper address subitem 1a-ii?

Copy and paste relevant sections from manuscript title (include quotes in quotation marks "like this" to indicate direct quotes from your manuscript), or elaborate on this item by providing additional information not in the ms, or briefly explain why the item is not applicable/relevant for your study

"Healthy Eating & Active Lifestyles for Diabetes (HEAL-D) is a co-designed culturally tailored DSMES programme for adults of black African and black Caribbean ethnicity. HEAL-D provides group-based education, behaviour change support and participatory physical activity sessions, delivered either face-to-face (F2F) or via online video conferencing."

"The programme is delivered by a diabetes specialist dietitian (no specified ethnicity), a community facilitator of black African and black Caribbean ethnicity, and exercise instructors (no specified ethnicity), using F2F or online video conferencing delivery modes"

1a-iii) Primary condition or target group in the title

Mention primary condition or target group in the title, if any (e.g., “for children with Type I Diabetes”) Example: A Web-based and Mobile Intervention with Telephone Support for Children with Type I Diabetes: Randomized Controlled Trial

	1	2	3	4	5	
subitem not at all important	○	○	○	○	◉	essential

Clear selection

Does your paper address subitem 1a-iii? *

Copy and paste relevant sections from manuscript title (include quotes in quotation marks "like this" to indicate direct quotes from your manuscript), or elaborate on this item by providing additional information not in the ms, or briefly explain why the item is not applicable/relevant for your study

"Healthy Eating & Active Lifestyles for Diabetes (HEAL-D): protocol for a multicentre, pragmatic randomised controlled trial of culturally tailored versus standard diabetes self-management education and support programmes in black African and black Caribbean adults with type 2 diabetes"

1b) ABSTRACT: Structured summary of trial design, methods, results, and conclusions

NPT extension: Description of experimental treatment, comparator, care providers, centers, and blinding status.

1b-i) Key features/functionalities/components of the intervention and comparator in the METHODS section of the ABSTRACT

Mention key features/functionalities/components of the intervention and comparator in the abstract. If possible, also mention theories and principles used for designing the site. Keep in mind the needs of systematic reviewers and indexers by including important synonyms. (Note: Only report in the abstract what the main paper is reporting. If this information is missing from the main body of text, consider adding it)

	1	2	3	4	5	
subitem not at all important	○	○	○	○	◉	essential

Clear selection

Does your paper address subitem 1b-i? *

Copy and paste relevant sections from the manuscript abstract (include quotes in quotation marks "like this" to indicate direct quotes from your manuscript), or elaborate on this item by providing additional information not in the ms, or briefly explain why the item is not applicable/relevant for your study

"Healthy Eating & Active Lifestyles for Diabetes (HEAL-D) is a co-designed culturally tailored DSMES programme for adults of black African and black Caribbean ethnicity. HEAL-D provides group-based education, behaviour change support and participatory physical activity sessions, delivered either face-to-face (F2F) or via online video conferencing. "

"Black African and black Caribbean adults with T2D (n=300), recruited from 3-5 centres in the UK (including London, West Midlands and Greater Manchester), will be randomised in a 1:1 ratio to HEAL-D (intervention) or a standard DSMES programme (control). "

1b-ii) Level of human involvement in the METHODS section of the ABSTRACT

Clarify the level of human involvement in the abstract, e.g., use phrases like “fully automated” vs. “therapist/nurse/care provider/physician-assisted” (mention number and expertise of providers involved, if any). (Note: Only report in the abstract what the main paper is reporting. If this information is missing from the main body of text, consider adding it)

	1	2	3	4	5	
subitem not at all important	○	◉	○	○	○	essential

Clear selection

Does your paper address subitem 1b-ii?

Copy and paste relevant sections from the manuscript abstract (include quotes in quotation marks "like this" to indicate direct quotes from your manuscript), or elaborate on this item by providing additional information not in the ms, or briefly explain why the item is not applicable/relevant for your study

"Baseline and follow-up visits (6, 12 and 24-months) will be conducted in person by research nurses for the measurement of HbA1c, blood lipids, anthropometric outcomes, blood pressure, physical activity, and patient reported outcome measures relating to psychological wellbeing and self-management support, lifestyle behaviours, and health economics."

1b-iii) Open vs. closed, web-based (self-assessment) vs. face-to-face assessments in the METHODS section of the ABSTRACT

Mention how participants were recruited (online vs. offline), e.g., from an open access website or from a clinic or a closed online user group (closed usergroup trial), and clarify if this was a purely web-based trial, or there were face-to-face components (as part of the intervention or for assessment). Clearly say if outcomes were self-assessed through questionnaires (as common in web-based trials). Note: In traditional offline trials, an open trial (open-label trial) is a type of clinical trial in which both the researchers and participants know which treatment is being administered. To avoid confusion, use “blinded” or “unblinded” to indicated the level of blinding instead of “open”, as “open” in web-based trials usually refers to “open access” (i.e. participants can self-enrol). (Note: Only report in the abstract what the main paper is reporting. If this information is missing from the main body of text, consider adding it)

	1	2	3	4	5	
subitem not at all important	○	◉	○	○	○	essential

Clear selection

Does your paper address subitem 1b-iii?

Copy and paste relevant sections from the manuscript abstract (include quotes in quotation marks "like this" to indicate direct quotes from your manuscript), or elaborate on this item by providing additional information not in the ms, or briefly explain why the item is not applicable/relevant for your study

"Baseline and follow-up visits (6, 12 and 24-months) will be conducted in person by research nurses for the measurement of HbA1c, blood lipids, anthropometric outcomes, blood pressure, physical activity, and patient reported outcome measures relating to psychological wellbeing and self-management support, lifestyle behaviours, and health economics."

1b-iv) RESULTS section in abstract must contain use data

Report number of participants enrolled/assessed in each group, the use/uptake of the intervention (e.g., attrition/adherence metrics, use over time, number of logins etc.), in addition to primary/secondary outcomes. (Note: Only report in the abstract what the main paper is reporting. If this information is missing from the main body of text, consider adding it)

	1	2	3	4	5	
subitem not at all important	(●)	( )	( )	( )	( )	essential

Clear selection

Does your paper address subitem 1b-iv?

Copy and paste relevant sections from the manuscript abstract (include quotes in quotation marks "like this" to indicate direct quotes from your manuscript), or elaborate on this item by providing additional information not in the ms, or briefly explain why the item is not applicable/relevant for your study

This is a protocol manuscript so there are no data to report at this stage.

1b-v) CONCLUSIONS/DISCUSSION in abstract for negative trials

Conclusions/Discussions in abstract for negative trials: Discuss the primary outcome - if the trial is negative (primary outcome not changed), and the intervention was not used, discuss whether negative results are attributable to lack of uptake and discuss reasons. (Note: Only report in the abstract what the main paper is reporting. If this information is missing from the main body of text, consider adding it)

	1	2	3	4	5	
subitem not at all important	(●)	( )	( )	( )	( )	essential

Clear selection

Does your paper address subitem 1b-v?

Copy and paste relevant sections from the manuscript abstract (include quotes in quotation marks "like this" to indicate direct quotes from your manuscript), or elaborate on this item by providing additional information not in the ms, or briefly explain why the item is not applicable/relevant for your study

This is a protocol manuscript so there are no data to report at this stage.

INTRODUCTION

2a) In INTRODUCTION: Scientific background and explanation of rationale

2a-i) Problem and the type of system/solution

Describe the problem and the type of system/solution that is object of the study: intended as stand-alone intervention vs. incorporated in broader health care program? Intended for a particular patient population? Goals of the intervention, e.g., being more cost-effective to other interventions, replace or complement other solutions? (Note: Details about the intervention are provided in “Methods” under 5)

	1	2	3	4	5	
subitem not at all important	○	○	○	○	◉	essential

Clear selection

Does your paper address subitem 2a-i? *

Copy and paste relevant sections from the manuscript (include quotes in quotation marks "like this" to indicate direct quotes from your manuscript), or elaborate on this item by providing additional information not in the ms, or briefly explain why the item is not applicable/relevant for your study

"The effects of T2D are experienced disproportionally by people of black African and black Caribbean ethnicity compared to white European ethnicity (7, 8), with 2-4 times greater prevalence (7, 8), younger onset (9), poorer outcomes for those who are affected, and greater medication requirements (10-12). A lack of access to T2D healthcare and reduced effectiveness of current T2D treatment programmes in black African and black Caribbean populations have been proposed as key causes for these inequalities (12-14). Therefore, the modification of existing T2D treatment pathways may facilitate better adoption and lower attrition rates in those of black African and black Caribbean ethnicity (15, 16), who make up the second largest and fastest growing UK minority ethnic group (17).
Optimising diet and physical activity and promoting self-management is an integral part of T2D management (18), with management guidelines recommending attendance at a diabetes self-management education and support (DSMES) programme (18). In the UK, quality standards require that DSMES programmes have an evidence-based curriculum, delivered by trained and competent educators, usually offering at least 6 hours of education (18). Several DSMES programmes are accredited and commissioned (19, 20), mainly using a group-based format and face-to-face (F2F) delivery, although digital programmes and online adaptations of F2F programmes have been evaluated or implemented following the COVID-19 pandemic (21, 22).
Whilst DSMES programmes are effective for improving T2D management and cardiovascular, behavioural and psychological outcomes, they are substantially less effective in people of black African and black Caribbean ethnicity (12, 15, 16). Despite recommendations for programmes to meet the needs of cultural groups (18), a lack of cultural knowledge and awareness among healthcare practitioners, as well as insensitivity to cultural beliefs and practices, are implicated as key drivers of this inequality (23-25).
Cultural tailoring of DSMES programmes to make them sensitive and responsive to health beliefs, practices and linguistic needs of cultural groups has been shown to enhance improvements in important outcomes, including glycaemic control (HbA1c), knowledge, and quality of life (25-27). However, few have been evaluated in the UK (28). Healthy Eating & Active Lifestyles for Diabetes (HEAL-D) is an evidence-based DSMES programme, tailored to the cultural needs of black African and black Caribbean adults, which was co-designed with black African and black Caribbean people living with T2D, healthcare practitioners and commissioners, and community leaders (29, 30). Patient acceptability of HEAL-D has been demonstrated in a feasibility trial (31) but it is not known if the intervention is clinically and cost effective. "

2a-ii) Scientific background, rationale: What is known about the (type of) system

Scientific background, rationale: What is known about the (type of) system that is the object of the study (be sure to discuss the use of similar systems for other conditions/diagnoses, if appropiate), motivation for the study, i.e. what are the reasons for and what is the context for this specific study, from which stakeholder viewpoint is the study performed, potential impact of findings [2]. Briefly justify the choice of the comparator.

	1	2	3	4	5	
subitem not at all important	○	○	○	◉	○	essential

Clear selection

Does your paper address subitem 2a-ii? *

Copy and paste relevant sections from the manuscript (include quotes in quotation marks "like this" to indicate direct quotes from your manuscript), or elaborate on this item by providing additional information not in the ms, or briefly explain why the item is not applicable/relevant for your study

"The effects of T2D are experienced disproportionally by people of black African and black Caribbean ethnicity compared to white European ethnicity (7, 8), with 2-4 times greater prevalence (7, 8), younger onset (9), poorer outcomes for those who are affected, and greater medication requirements (10-12). A lack of access to T2D healthcare and reduced effectiveness of current T2D treatment programmes in black African and black Caribbean populations have been proposed as key causes for these inequalities (12-14). Therefore, the modification of existing T2D treatment pathways may facilitate better adoption and lower attrition rates in those of black African and black Caribbean ethnicity (15, 16), who make up the second largest and fastest growing UK minority ethnic group (17).
Optimising diet and physical activity and promoting self-management is an integral part of T2D management (18), with management guidelines recommending attendance at a diabetes self-management education and support (DSMES) programme (18). In the UK, quality standards require that DSMES programmes have an evidence-based curriculum, delivered by trained and competent educators, usually offering at least 6 hours of education (18). Several DSMES programmes are accredited and commissioned (19, 20), mainly using a group-based format and face-to-face (F2F) delivery, although digital programmes and online adaptations of F2F programmes have been evaluated or implemented following the COVID-19 pandemic (21, 22).
Whilst DSMES programmes are effective for improving T2D management and cardiovascular, behavioural and psychological outcomes, they are substantially less effective in people of black African and black Caribbean ethnicity (12, 15, 16). Despite recommendations for programmes to meet the needs of cultural groups (18), a lack of cultural knowledge and awareness among healthcare practitioners, as well as insensitivity to cultural beliefs and practices, are implicated as key drivers of this inequality (23-25). Cultural tailoring of DSMES programmes to make them sensitive and responsive to health beliefs, practices and linguistic needs of cultural groups has been shown to enhance improvements in important outcomes, including glycaemic control (HbA1c), knowledge, and quality of life (25-27). However, few have been evaluated in the UK (28). Healthy Eating & Active Lifestyles for Diabetes (HEAL-D) is an evidence-based DSMES programme, tailored to the cultural needs of black African and black Caribbean adults, which was co-designed with black African and black Caribbean people living with T2D, healthcare practitioners and commissioners, and community leaders (29, 30). Patient acceptability of HEAL-D has been demonstrated in a feasibility trial (31) but it is not known if the intervention is clinically and cost effective. "

2b) In INTRODUCTION: Specific objectives or hypotheses

Does your paper address CONSORT subitem 2b? *

Copy and paste relevant sections from the manuscript (include quotes in quotation marks "like this" to indicate direct quotes from your manuscript), or elaborate on this item by providing additional information not in the ms, or briefly explain why the item is not applicable/relevant for your study

"The primary aim of this trial is to evaluate the effectiveness of the HEAL-D intervention, compared to standard DSMES programmes, on glycaemic control (assessed via HbA1c) at 12-months in black African and black Caribbean adults living with T2D. It is hypothesised that the HEAL-D intervention will improve glycaemic control to a greater extent than standard DSMES programmes at 12 months follow-up. Secondary aims include testing the effectiveness of HEAL-D, compared to standard DSMES programmes, on: cardiovascular risk factors; psychological wellbeing and quality of life; T2D knowledge and self-efficacy; and diet and physical activity behaviours at 6, 12 and 24 months, as well as assessing cost-effectiveness. A mixed methods process evaluation aims to assess HEAL-D delivery, intervention fidelity and implementation, and the impact of multiple long-term conditions (MLTC) on recruitment and engagement with the HEAL-D intervention, and the impact of HEAL-D on MLTC. "

METHODS

3a) Description of trial design (such as parallel, factorial) including allocation ratio

Does your paper address CONSORT subitem 3a? *

Copy and paste relevant sections from the manuscript (include quotes in quotation marks "like this" to indicate direct quotes from your manuscript), or elaborate on this item by providing additional information not in the ms, or briefly explain why the item is not applicable/relevant for your study

"The trial is a 24-month, multi-centre, pragmatic, open-label, 2-arm, parallel-group, individually randomised group treatment trial"

3b) Important changes to methods after trial commencement (such as eligibility criteria), with reasons

Does your paper address CONSORT subitem 3b? *

Copy and paste relevant sections from the manuscript (include quotes in quotation marks "like this" to indicate direct quotes from your manuscript), or elaborate on this item by providing additional information not in the ms, or briefly explain why the item is not applicable/relevant for your study

This is a protocol manuscript so there are no amendments to report at this stage.

3b-i) Bug fixes, Downtimes, Content Changes

Bug fixes, Downtimes, Content Changes: ehealth systems are often dynamic systems. A description of changes to methods therefore also includes important changes made on the intervention or comparator during the trial (e.g., major bug fixes or changes in the functionality or content) (5-iii) and other “unexpected events” that may have influenced study design such as staff changes, system failures/downtimes, etc. [2].

	1	2	3	4	5	
subitem not at all important	●	○	○	○	○	essential

Clear selection

Does your paper address subitem 3b-i?

Copy and paste relevant sections from the manuscript (include quotes in quotation marks "like this" to indicate direct quotes from your manuscript), or elaborate on this item by providing additional information not in the ms, or briefly explain why the item is not applicable/relevant for your study

This is a protocol manuscript so there are none to report at this stage.

4a) Eligibility criteria for participants

Does your paper address CONSORT subitem 4a? *

Copy and paste relevant sections from the manuscript (include quotes in quotation marks "like this" to indicate direct quotes from your manuscript), or elaborate on this item by providing additional information not in the ms, or briefly explain why the item is not applicable/relevant for your study

"Inclusion criteria are:
• Adult (≥18 years of age).
• Black African or black Caribbean ethnicity (self-declared and classified according to the Census system used within NHS services: people of 'black African', 'black Caribbean', 'black British', 'black other', and 'mixed race' with either African or Caribbean ancestry).
• T2D diagnosis (confirmed by medical history).
• HbA1c ≤100 mmol/mol (or fructosamine <450 μmol for individuals with sickle cell trait/disease).
• Suitable for group-based training and participation in physical activity (suitability confirmed by a general practitioner [GP] or referring healthcare practitioner).
• Willing to undergo randomisation.
• Able to provide informed consent.
Exclusion criteria are:
• Current pregnancy.
• Complex medical, lifestyle or learning needs that require personalised advice or for which group-based training is unsuitable (e.g., advanced chronic kidney disease, people with learning disabilities; confirmed by GP or referring healthcare practitioner).
• Need for language translation services (spoken or written).
• Unable/unwilling to provided informed consent.
• Current participation in competing clinical trial (as determined by trial investigator).
The eligibility criteria have been selected pragmatically to align with UK clinical practice, whereby group-based DSMES is a recommended management option for most patients with T2D. However, patients with uncontrolled T2D (i.e. high HbA1c), or specific/complex medical, educational, lifestyle needs, would not typically be referred to group-based DSMES programmes, but would be managed in services that can provide more individualised advice/care. Similarly, patients needing language translation services would not typically be referred to group-based DSMES programmes because of difficulties meeting their language needs."

4a-i) Computer / Internet literacy

Computer / Internet literacy is often an implicit “de facto” eligibility criterion - this should be explicitly clarified.

	1	2	3	4	5	
subitem not at all important	(●)	○	○	○	○	essential

Clear selection

Does your paper address subitem 4a-i?

Copy and paste relevant sections from the manuscript (include quotes in quotation marks "like this" to indicate direct quotes from your manuscript), or elaborate on this item by providing additional information not in the ms, or briefly explain why the item is not applicable/relevant for your study

Participants are given a choice of which delivery mode they wish to attend so there is no requirement for them to have digital literacy.

4a-ii) Open vs. closed, web-based vs. face-to-face assessments:

Open vs. closed, web-based vs. face-to-face assessments: Mention how participants were recruited (online vs. offline), e.g., from an open access website or from a clinic, and clarify if this was a purely web-based trial, or there were face-to-face components (as part of the intervention or for assessment), i.e., to what degree got the study team to know the participant. In online-only trials, clarify if participants were quasi-anonymous and whether having multiple identities was possible or whether technical or logistical measures (e.g., cookies, email confirmation, phone calls) were used to detect/prevent these.

	1	2	3	4	5	
subitem not at all important	(●)	○	○	○	○	essential

Clear selection

Does your paper address subitem 4a-ii? *

Copy and paste relevant sections from the manuscript (include quotes in quotation marks "like this" to indicate direct quotes from your manuscript), or elaborate on this item by providing additional information not in the ms, or briefly explain why the item is not applicable/relevant for your study

"The measures will be collected during an in-person assessment of approximately 2 hours duration at an NHS research or primary care clinic facility."

4a-iii) Information giving during recruitment

Information given during recruitment. Specify how participants were briefed for recruitment and in the informed consent procedures (e.g., publish the informed consent documentation as appendix, see also item X26), as this information may have an effect on user self-selection, user expectation and may also bias results.

	1	2	3	4	5	
subitem not at all important	○	○	○	○	●	essential

Clear selection

Does your paper address subitem 4a-iii?

Copy and paste relevant sections from the manuscript (include quotes in quotation marks "like this" to indicate direct quotes from your manuscript), or elaborate on this item by providing additional information not in the ms, or briefly explain why the item is not applicable/relevant for your study

"The research team will work with healthcare providers and grassroots/community organisations to identify and recruit participants. Several means of recruitment will be utilised:
[i] screening of referrals to DSMES services from primary, intermediate/community and secondary care clinics;
[ii] primary care database searches;
[iii] self-referral in response to study advertisements and community engagement activities.
Potential participants will be sent a participant information video via text message, or a written information leaflet via email or letter, providing a brief explanation of the purpose of the trial and participation requirements. Interested individuals will be invited to contact the research team or a community champion by phone, email or web-form to discuss the trial in detail and schedule a telephone eligibility screening, which will be conducted by the research team. Eligible participants, who confirm their intention to participate, will be invited to attend an in-person visit where written informed consent will be recorded prior to baseline data collection."

4b) Settings and locations where the data were collected

Does your paper address CONSORT subitem 4b? *

Copy and paste relevant sections from the manuscript (include quotes in quotation marks "like this" to indicate direct quotes from your manuscript), or elaborate on this item by providing additional information not in the ms, or briefly explain why the item is not applicable/relevant for your study

"The trial will be conducted in 3-5 centres in the UK, including London, West Midlands and Greater Manchester, which have been selected as areas in the UK with significant representation of black African and black Caribbean adults. "

4b-i) Report if outcomes were (self-)assessed through online questionnaires

Clearly report if outcomes were (self-)assessed through online questionnaires (as common in web-based trials) or otherwise.

	1	2	3	4	5	
subitem not at all important	○	○	◉	○	○	essential

Clear selection

Does your paper address subitem 4b-i? *

Copy and paste relevant sections from the manuscript (include quotes in quotation marks "like this" to indicate direct quotes from your manuscript), or elaborate on this item by providing additional information not in the ms, or briefly explain why the item is not applicable/relevant for your study

"The questionnaires will be interviewer-led or self-complete depending on participant preference and/or literacy. "

4b-ii) Report how institutional affiliations are displayed

Report how institutional affiliations are displayed to potential participants [on ehealth media], as affiliations with prestigious hospitals or universities may affect volunteer rates, use, and reactions with regards to an intervention.(Not a required item – describe only if this may bias results)

	1	2	3	4	5	
subitem not at all important	◉	○	○	○	○	essential

Clear selection

Does your paper address subitem 4b-ii?

Copy and paste relevant sections from the manuscript (include quotes in quotation marks "like this" to indicate direct quotes from your manuscript), or elaborate on this item by providing additional information not in the ms, or briefly explain why the item is not applicable/relevant for your study

Your answer

5) The interventions for each group with sufficient details to allow replication, including how and when they were actually administered

5-i) Mention names, credential, affiliations of the developers, sponsors, and owners

Mention names, credential, affiliations of the developers, sponsors, and owners [6] (if authors/evaluators are owners or developer of the software, this needs to be declared in a "Conflict of interest" section or mentioned elsewhere in the manuscript).

	1	2	3	4	5	
subitem not at all important	○	○	◉	○	○	essential

Clear selection

Does your paper address subitem 5-i?

Copy and paste relevant sections from the manuscript (include quotes in quotation marks "like this" to indicate direct quotes from your manuscript), or elaborate on this item by providing additional information not in the ms, or briefly explain why the item is not applicable/relevant for your study

There are no commercial developers, sponsors or owners.

5-ii) Describe the history/development process

Describe the history/development process of the application and previous formative evaluations (e.g., focus groups, usability testing), as these will have an impact on adoption/use rates and help with interpreting results.

	1	2	3	4	5	
subitem not at all important	○	○	◉	○	○	essential

Clear selection

Does your paper address subitem 5-ii?

Copy and paste relevant sections from the manuscript (include quotes in quotation marks "like this" to indicate direct quotes from your manuscript), or elaborate on this item by providing additional information not in the ms, or briefly explain why the item is not applicable/relevant for your study

"The HEAL-D intervention was developed in an earlier programme of research funded by the National Institute for Health Research (NIHR); the intervention development process and findings and evaluation of patient acceptability have been published previously."

5-iii) Revisions and updating

Revisions and updating. Clearly mention the date and/or version number of the application/intervention (and comparator, if applicable) evaluated, or describe whether the intervention underwent major changes during the evaluation process, or whether the development and/or content was "frozen" during the trial. Describe dynamic components such as news feeds or changing content which may have an impact on the replicability of the intervention (for unexpected events see item 3b).

	1	2	3	4	5	
subitem not at all important	◉	○	○	○	○	essential

Clear selection

Does your paper address subitem 5-iii?

Copy and paste relevant sections from the manuscript (include quotes in quotation marks "like this" to indicate direct quotes from your manuscript), or elaborate on this item by providing additional information not in the ms, or briefly explain why the item is not applicable/relevant for your study

This is a protocol manuscript so this item is not relevant at this stage.

5-iv) Quality assurance methods

Provide information on quality assurance methods to ensure accuracy and quality of information provided [1], if applicable.

	1	2	3	4	5	
subitem not at all important	○	○	●	○	○	essential

Clear selection

Does your paper address subitem 5-iv?

Copy and paste relevant sections from the manuscript (include quotes in quotation marks "like this" to indicate direct quotes from your manuscript), or elaborate on this item by providing additional information not in the ms, or briefly explain why the item is not applicable/relevant for your study

"Implementation will encompass: fidelity (whether training and the intervention sessions were delivered as intended, whether healthcare professionals delivered culturally sensitive behaviour change support and any observed barriers to this), intervention dose (i.e., attendance and completion rates), and reach (whether the intended audience encounters the intervention, and how). "

5-v) Ensure replicability by publishing the source code, and/or providing screenshots/screen-capture video, and/or providing flowcharts of the algorithms used

Ensure replicability by publishing the source code, and/or providing screenshots/screen-capture video, and/or providing flowcharts of the algorithms used. Replicability (i.e., other researchers should in principle be able to replicate the study) is a hallmark of scientific reporting.

	1	2	3	4	5	
subitem not at all important	◉	○	○	○	○	essential

Clear selection

Does your paper address subitem 5-v?

Copy and paste relevant sections from the manuscript (include quotes in quotation marks "like this" to indicate direct quotes from your manuscript), or elaborate on this item by providing additional information not in the ms, or briefly explain why the item is not applicable/relevant for your study

No algorithms used in this research

5-vi) Digital preservation

Digital preservation: Provide the URL of the application, but as the intervention is likely to change or disappear over the course of the years; also make sure the intervention is archived (Internet Archive, webcitation.org, and/or publishing the source code or screenshots/videos alongside the article). As pages behind login screens cannot be archived, consider creating demo pages which are accessible without login.

	1	2	3	4	5	
subitem not at all important	◉	○	○	○	○	essential

Clear selection

Does your paper address subitem 5-vi?

Copy and paste relevant sections from the manuscript (include quotes in quotation marks "like this" to indicate direct quotes from your manuscript), or elaborate on this item by providing additional information not in the ms, or briefly explain why the item is not applicable/relevant for your study

The intervention uses video conferencing rather than an application

5-vii) Access

Access: Describe how participants accessed the application, in what setting/context, if they had to pay (or were paid) or not, whether they had to be a member of specific group. If known, describe how participants obtained “access to the platform and Internet” [1]. To ensure access for editors/reviewers/readers, consider to provide a “backdoor” login account or demo mode for reviewers/readers to explore the application (also important for archiving purposes, see vi).

	1	2	3	4	5	
subitem not at all important	(●)	( )	( )	( )	( )	essential

Clear selection

Does your paper address subitem 5-vii? *

Copy and paste relevant sections from the manuscript (include quotes in quotation marks "like this" to indicate direct quotes from your manuscript), or elaborate on this item by providing additional information not in the ms, or briefly explain why the item is not applicable/relevant for your study

The intervention uses video conferencing rather than an application

5-viii) Mode of delivery, features/functionalities/components of the intervention and comparator, and the theoretical framework

Describe mode of delivery, features/functionalities/components of the intervention and comparator, and the theoretical framework [6] used to design them (instructional strategy [1], behaviour change techniques, persuasive features, etc., see e.g., [7, 8] for terminology). This includes an in-depth description of the content (including where it is coming from and who developed it) [1]," whether [and how] it is tailored to individual circumstances and allows users to track their progress and receive feedback" [6]. This also includes a description of communication delivery channels and – if computer-mediated communication is a component – whether communication was synchronous or asynchronous [6]. It also includes information on presentation strategies [1], including page design principles, average amount of text on pages, presence of hyperlinks to other resources, etc. [1].

	1	2	3	4	5	
subitem not at all important	○	○	◉	○	○	essential

Clear selection

Does your paper address subitem 5-viii? *

Copy and paste relevant sections from the manuscript (include quotes in quotation marks "like this" to indicate direct quotes from your manuscript), or elaborate on this item by providing additional information not in the ms, or briefly explain why the item is not applicable/relevant for your study

"HEAL-D aligns with quality standards (18), is underpinned by an evidence-based curriculum (37), and uses evidence-based behaviour change techniques (BCTs), informed by the Behaviour Change Wheel and the Capability Opportunity Motivation-Behaviour (COM-B) framework (38), to support adoption and long-term maintenance of the following diet and lifestyle goals (37)"

5-ix) Describe use parameters

Describe use parameters (e.g., intended “doses” and optimal timing for use). Clarify what instructions or recommendations were given to the user, e.g., regarding timing, frequency, heaviness of use, if any, or was the intervention used ad libitum.

	1	2	3	4	5	
subitem not at all important	○	○	●	○	○	essential

Clear selection

Does your paper address subitem 5-ix?

Copy and paste relevant sections from the manuscript (include quotes in quotation marks "like this" to indicate direct quotes from your manuscript), or elaborate on this item by providing additional information not in the ms, or briefly explain why the item is not applicable/relevant for your study

"HEAL-D is a culturally tailored DSMES programme, consisting of 16 hours (eight 2-hr sessions delivered over 6 months) of group-based T2D self-management education and support, including participatory physical activity classes."

5-x) Clarify the level of human involvement

Clarify the level of human involvement (care providers or health professionals, also technical assistance) in the e-intervention or as co-intervention (detail number and expertise of professionals involved, if any, as well as “type of assistance offered, the timing and frequency of the support, how it is initiated, and the medium by which the assistance is delivered”. It may be necessary to distinguish between the level of human involvement required for the trial, and the level of human involvement required for a routine application outside of a RCT setting (discuss under item 21 – generalizability).

	1	2	3	4	5	
subitem not at all important	○	○	○	●	○	essential

Clear selection

Does your paper address subitem 5-x?

Copy and paste relevant sections from the manuscript (include quotes in quotation marks "like this" to indicate direct quotes from your manuscript), or elaborate on this item by providing additional information not in the ms, or briefly explain why the item is not applicable/relevant for your study

"The programme is delivered by a diabetes specialist dietitian (no specified ethnicity), a community facilitator of black African and black Caribbean ethnicity, and exercise instructors (no specified ethnicity), using F2F or online video conferencing delivery modes; participants will choose their mode of attendance. "

5-xi) Report any prompts/reminders used

Report any prompts/reminders used: Clarify if there were prompts (letters, emails, phone calls, SMS) to use the application, what triggered them, frequency etc. It may be necessary to distinguish between the level of prompts/reminders required for the trial, and the level of prompts/reminders for a routine application outside of a RCT setting (discuss under item 21 – generalizability).

	1	2	3	4	5	
subitem not at all important	(●)	( )	( )	( )	( )	essential

Clear selection

Does your paper address subitem 5-xi? *

Copy and paste relevant sections from the manuscript (include quotes in quotation marks "like this" to indicate direct quotes from your manuscript), or elaborate on this item by providing additional information not in the ms, or briefly explain why the item is not applicable/relevant for your study

There are no applications used, no prompts

5-xii) Describe any co-interventions (incl. training/support)

Describe any co-interventions (incl. training/support): Clearly state any interventions that are provided in addition to the targeted eHealth intervention, as ehealth intervention may not be designed as stand-alone intervention. This includes training sessions and support [1]. It may be necessary to distinguish between the level of training required for the trial, and the level of training for a routine application outside of a RCT setting (discuss under item 21 – generalizability.

	1	2	3	4	5	
subitem not at all important	(•)	○	○	○	○	essential

Clear selection

Does your paper address subitem 5-xii? *

Copy and paste relevant sections from the manuscript (include quotes in quotation marks "like this" to indicate direct quotes from your manuscript), or elaborate on this item by providing additional information not in the ms, or briefly explain why the item is not applicable/relevant for your study

There are no co-interventions involved

6a) Completely defined pre-specified primary and secondary outcome measures, including how and when they were assessed

Does your paper address CONSORT subitem 6a? *

Copy and paste relevant sections from the manuscript (include quotes in quotation marks "like this" to indicate direct quotes from your manuscript), or elaborate on this item by providing additional information not in the ms, or briefly explain why the item is not applicable/relevant for your study

"The primary outcome upon which the trial is powered is difference between groups in the change in HbA1c from baseline to 12-months. HbA1c was chosen for several reasons: it is the principal clinical measure of diabetes status and glycaemic control, and a valuable surrogate measure of holistic engagement with diabetes management and self-care; reduction of HbA1c is associated with reduced risk of micro- and macro-vascular complications and, in some cases, all-cause mortality (39); it is also a prominent component of the Core Outcome Measures in Effectiveness Trials (COMET) initiative core outcomes set for T2D (40). A 12-month primary endpoint has been chosen to examine the effectiveness of the HEAL-D intervention as this is a duration long enough to observe a clinically important difference in HbA1c.
Secondary and Exploratory Outcomes
At 6 and 24 months, HbA1c will be measured as a secondary outcome to allow exploration of the time-course of any observed changes and impact of HEAL-D over a longer period.
Other secondary outcomes (Table 1), measured at 6, 12 and 24 months, are grouped into holistic health domains and include: cardiovascular risk factors; psychological wellbeing and self-management support; lifestyle behaviours; and health economics.
Tertiary outcomes are: low-density lipoprotein (LDL)-cholesterol, triglycerides, body fat percentage, changes to glucose-lowering and anti-hypertensive therapies (including addition, removal or dose adjustment), and change in MLTC status (including additional diagnoses, remission, or changes in severity).
Measurements
At baseline and all follow-up visits, unless specified otherwise, the following measurements will be conducted.
HbA1c and blood lipids
A 5mL venous blood sample (non-fasting) will be taken via venepuncture according to local standardised operating procedures (SOPs) for measurement of HbA1c (EDTA tube) and full lipid profile (gel-activated clotting agent tube) by the pathology department at the corresponding clinical site. Biological samples taken for the study will be destroyed once analysed in accordance with the Human Tissue Act 2004.
Blood pressure
Brachial arterial blood pressure will be measured in the seated position using an automated sphygmomanometer after participants have been resting for ~5 minutes. Three blood pressure measurements will be obtained and the average of the last two measurements will be used.
Anthropometry
Body weight will be measured using digital scales, with the patient wearing light clothing (without shoes), to the nearest 0.1 kg. Height will be measured to the nearest 0.5 cm, using a stadiometer, without shoes. Waist circumference will be measured using a flexible tape, with the patient wearing only light clothing, using the WHO methodology, which defines the 'waist' as the mid-point between the lowest rib and the iliac crest. The mean of three waist circumference measurements will be recorded. Body composition will be measured using Tanita DC-430-MA P bioelectrical impedance scales; body fat (%) and lean mass (kg) will be recorded.
Physical activity
Physical activity will be measured objectively, using a wrist-worn accelerometer over 7-10 days, measuring sleep, inactivity, step count and moderate-to-vigorous physical activity. Accelerometer use will occur at baseline and the 12-month visits, only. Self-reported physical activity will be recorded using the short International Physical Activity Questionnaire (s-IPAQ), which consists of seven questions about the amount of time spent in different levels of physical activity (vigorous, moderate and low intensity), categorising overall physical activity levels as low, moderate or high (REF).
Patient reported outcome measures

[illegible]
Several patient reported outcome measures, including diabetes-specific measures, will be collected:
• Quality of life (QoL) will be assessed using EuroQol-5 Dimensions-5 Levels (EQ5D-5L) (REF) and used in the cost effectiveness evaluation.
• Diabetes-related distress will be assessed using the 5-point Problem Areas In Diabetes (PAID-5) questionnaire (REF); PAID-5 is widely used in diabetes trials as an indicator of diabetes-specific QoL.
• Depressive symptoms will be assessed using the Patient Health Questionnaire (PHQ-9) (REF); PHQ-9 is used widely, including in the NHS, to assess symptoms of depression.
• Diabetes knowledge will be assessed using the Short Diabetes Knowledge Instrument (SDKI) (REF).
• Diabetes self-efficacy will be assessed using the Diabetes Management Self-Efficacy Scale (DMSES-UK) (REF).
• Diabetes dietary competence will be assessed using the Perceived Diabetes & Dietary Competence (PDDC) (REF).
• Multimorbidity treatment burden will be assessed using the Multimorbidity Treatment Burden Questionnaire (MTBQ) (REF).
• The Diet Quality Questionnaire (DQQ) will be used to assess dietary adequacy, providing a measure of diet diversity and protection against non-communicable diseases (REF).
• Health service resource utilisation will be assessed using an adapted Adult Service Use Schedule (AD-SUS) (REF), and will be used to inform the health economic evaluation.
• Self-reported sleep duration and chronotype will be assessed using XXX.
The questionnaires have evidenced reliability and validity for use in people with T2D (REF); none of the questionnaires have been validated specifically in populations of African and Caribbean heritage, although the SDKI and PDDC have been used in studies with African-American populations. The questionnaires will be interviewer-led or self-complete depending on participant preference and/or literacy. "

6a-i) Online questionnaires: describe if they were validated for online use and apply CHERRIES items to describe how the questionnaires were designed/deployed

If outcomes were obtained through online questionnaires, describe if they were validated for online use and apply CHERRIES items to describe how the questionnaires were designed/deployed [9].

	1	2	3	4	5	
subitem not at all important	◉	○	○	○	○	essential

Clear selection

Does your paper address subitem 6a-i?

Copy and paste relevant sections from manuscript text

There are no online questionnaires included

6a-ii) Describe whether and how “use” (including intensity of use/dosage) was defined/measured/monitored

Describe whether and how “use” (including intensity of use/dosage) was defined/measured/monitored (logins, logfile analysis, etc.). Use/adoption metrics are important process outcomes that should be reported in any ehealth trial.

	1	2	3	4	5	
subitem not at all important	◉	○	○	○	○	essential

Clear selection

Does your paper address subitem 6a-ii?

Copy and paste relevant sections from manuscript text

Not included in the trial

6a-iii) Describe whether, how, and when qualitative feedback from participants was obtained

Describe whether, how, and when qualitative feedback from participants was obtained (e.g., through emails, feedback forms, interviews, focus groups).

	1	2	3	4	5	
subitem not at all important	○	○	○	◉	○	essential

Clear selection

Does your paper address subitem 6a-iii?

Copy and paste relevant sections from manuscript text

"An embedded mixed methods process evaluation, combining relevant data gathered within the trial (questionnaires, monitoring data), qualitative interview data, logbook entries, workshops and observations will provide a formative evaluation of the intervention's implementation, mechanisms of action, and identification of the contextual factors that influence its implementation and adoption. Implementation will encompass: fidelity (whether training and the intervention sessions were delivered as intended, whether healthcare professionals delivered culturally sensitive behaviour change support and any observed barriers to this), intervention dose (i.e., attendance and completion rates), and reach (whether the intended audience encounters the intervention, and how). Mechanisms of action will encompass: satisfaction (with the programme), participant and facilitator experience of the intervention, and effective and less effective components of the intervention in engaging participants and producing desired results (e.g., increasing physical activity) and potential reasons. The identification of contextual factors that influence the implementation and adoption of HEAL-D includes implications for workforce capacity and for intervention integration within existing care.
Participants who are randomised to the HEAL-D intervention arm and staff involved in HEAL-D delivery will be eligible to participate in the process evaluation. We aim to recruit a total of 48 participants (16 per centre) based on sampling for diversity in relation to high vs low intervention engagement, F2F vs online attendance, and MLTC vs non-MLTC status. Maximum variation in sampling of participants will be guided by age, gender, employment status, and ethnicity (black African or black Caribbean). The characteristics of the sample will be continually reviewed to achieve balanced representation, inviting consecutive participants until the target sample and data saturation has been achieved. We aim to recruit up to 27 staff (9 per centre), including delivery staff and trainers. Staff must meet the following criteria to participate: clinical and non-clinical staff working at/in partnership with centres participating in the HEAL-D trial, age ≥18 years, and able to give written informed consent.
Data for the process evaluation will be collected via several means. Firstly, both the training of HEAL-D facilitators and the delivery of HEAL-D sessions by facilitators will be observed. A self-report questionnaire, completed at the 12-month visit, will be used to assess acceptability of trial procedures by participants. Participants will also be invited to undertake a semi-structured interviews within one month after the 12-month visit. These interviews will be conducted by telephone and provide an open and flexible method for exploring individual experiences in-depth. Participants and staff will also be invited to attend a F2F workshop to explore HEAL-D implementation.
"

6b) Any changes to trial outcomes after the trial commenced, with reasons

Does your paper address CONSORT subitem 6b? *

Copy and paste relevant sections from the manuscript (include quotes in quotation marks "like this" to indicate direct quotes from your manuscript), or elaborate on this item by providing additional information not in the ms, or briefly explain why the item is not applicable/relevant for your study

This is a protocol manuscript so this item is not relevant at present

7a) How sample size was determined

NPT: When applicable, details of whether and how the clustering by care provides or centers was addressed

7a-i) Describe whether and how expected attrition was taken into account when calculating the sample size

Describe whether and how expected attrition was taken into account when calculating the sample size.

	1	2	3	4	5	
subitem not at all important	○	○	○	○	◉	essential

Clear selection

Does your paper address subitem 7a-i?

Copy and paste relevant sections from manuscript title (include quotes in quotation marks "like this" to indicate direct quotes from your manuscript), or elaborate on this item by providing additional information not in the ms, or briefly explain why the item is not applicable/relevant for your study

"The trial is based on a minimal clinically important difference in HbA1c of 5 mmol/mol, as leading to significant risk reductions for T2D complications (3). Power was calculated at 90%, with a 5% 2-sided significance level, to detect a standardised effect size of 0.45 (difference in HbA1c of 5 and standard deviation of 11 mmol/mol, determined from the feasibility trial (31) and unpublished primary care data from south London). To allow for correlation of outcomes among group attendees the sample size assuming no correlation (103 per arm) is inflated by a design effect (1.09) and then rounded up to ensure divisibility by group size. The F2F intervention is to be delivered in groups of up to 12, while online intervention will be delivered in groups of up to 8; for the calculations an average group size of 10 is assumed. Given the objective outcome, short duration of treatment, patterns observed in the intracluster correlation coefficient (ICC) for cluster randomised trials suggest an ICC of 0.01 (46). In the previous feasibility trial, loss to follow-up was 7% at 6 months. Given the pragmatic design, whereby participants are given free choice as to their mode of attendance (F2F or online) in both the intervention and comparator arms, we do not expect substantial differences in retention between arms. However, with a longer primary outcome follow-up of 12 months, loss to follow-up is estimated to be higher at 15%. For the intervention arm, this is accounted for by increasing the number of clusters. Therefore, we will recruit 150 participants in the control arm and 150 participants in the intervention arm, each across 15 groups of average size 10."

7b) When applicable, explanation of any interim analyses and stopping guidelines

Does your paper address CONSORT subitem 7b? *

Copy and paste relevant sections from the manuscript (include quotes in quotation marks "like this" to indicate direct quotes from your manuscript), or elaborate on this item by providing additional information not in the ms, or briefly explain why the item is not applicable/relevant for your study

"A feasibility assessment will be conducted in the first 6 months of the trial to establish the feasibility of completing the trial within desired timelines, focused on (1) identification of eligible participants, (2) consent and randomisation of eligible participants, and (3) engagement with treatment allocation of randomised participants. Recruitment and intervention engagement data will be reviewed by the TSC with predefined ‘stop/go’ criteria to determine progression to full trial."

8a) Method used to generate the random allocation sequence
NPT: When applicable, how care providers were allocated to each trial group

Does your paper address CONSORT subitem 8a? *

Copy and paste relevant sections from the manuscript (include quotes in quotation marks "like this" to indicate direct quotes from your manuscript), or elaborate on this item by providing additional information not in the ms, or briefly explain why the item is not applicable/relevant for your study

"Following the baseline assessment, each participant will be randomly allocated (1:1 ratio) to the intervention or control group using a centralised web-based randomisation database (REDCap). Allocation will use randomly permuted blocks of variable block length and be stratified by centre, accounting for provision of different standard DSMES programmes between centres, and baseline HbA1c (48-52, 53-76, 77-100 mmol/mol). The participants and researchers will not be blinded to group allocation. "

8b) Type of randomisation; details of any restriction (such as blocking and block size)

Does your paper address CONSORT subitem 8b? *

Copy and paste relevant sections from the manuscript (include quotes in quotation marks "like this" to indicate direct quotes from your manuscript), or elaborate on this item by providing additional information not in the ms, or briefly explain why the item is not applicable/relevant for your study

"Following the baseline assessment, each participant will be randomly allocated (1:1 ratio) to the intervention or control group using a centralised web-based randomisation database (REDCap). Allocation will use randomly permuted blocks of variable block length and be stratified by centre, accounting for provision of different standard DSMES programmes between centres, and baseline HbA1c (48-52, 53-76, 77-100 mmol/mol). The participants and researchers will not be blinded to group allocation. "

9) Mechanism used to implement the random allocation sequence (such as sequentially numbered containers), describing any steps taken to conceal the sequence until interventions were assigned

Does your paper address CONSORT subitem 9? *

Copy and paste relevant sections from the manuscript (include quotes in quotation marks "like this" to indicate direct quotes from your manuscript), or elaborate on this item by providing additional information not in the ms, or briefly explain why the item is not applicable/relevant for your study

"Following the baseline assessment, each participant will be randomly allocated (1:1 ratio) to the intervention or control group using a centralised web-based randomisation database (REDCap). Allocation will use randomly permuted blocks of variable block length and be stratified by centre, accounting for provision of different standard DSMES programmes between centres, and baseline HbA1c (48-52, 53-76, 77-100 mmol/mol). The participants and researchers will not be blinded to group allocation. "

10) Who generated the random allocation sequence, who enrolled participants, and who assigned participants to interventions

Does your paper address CONSORT subitem 10? *

Copy and paste relevant sections from the manuscript (include quotes in quotation marks "like this" to indicate direct quotes from your manuscript), or elaborate on this item by providing additional information not in the ms, or briefly explain why the item is not applicable/relevant for your study

"Following the baseline assessment, each participant will be randomly allocated (1:1 ratio) to the intervention or control group using a centralised web-based randomisation database (REDCap). Allocation will use randomly permuted blocks of variable block length and be stratified by centre, accounting for provision of different standard DSMES programmes between centres, and baseline HbA1c (48-52, 53-76, 77-100 mmol/mol). The participants and researchers will not be blinded to group allocation. "

11a) If done, who was blinded after assignment to interventions (for example, participants, care providers, those assessing outcomes) and how

NPT: Whether or not administering co-interventions were blinded to group assignment

11a-i) Specify who was blinded, and who wasn’t

Specify who was blinded, and who wasn’t. Usually, in web-based trials it is not possible to blind the participants [1, 3] (this should be clearly acknowledged), but it may be possible to blind outcome assessors, those doing data analysis or those administering co-interventions (if any).

	1	2	3	4	5	
subitem not at all important	○	○	○	◉	○	essential

Clear selection

Does your paper address subitem 11a-i? *

Copy and paste relevant sections from the manuscript (include quotes in quotation marks "like this" to indicate direct quotes from your manuscript), or elaborate on this item by providing additional information not in the ms, or briefly explain why the item is not applicable/relevant for your study

"The participants and researchers will not be blinded to group allocation. "

11a-ii) Discuss e.g., whether participants knew which intervention was the “intervention of interest” and which one was the “comparator”

Informed consent procedures (4a-ii) can create biases and certain expectations - discuss e.g., whether participants knew which intervention was the “intervention of interest” and which one was the “comparator”.

	1	2	3	4	5	
subitem not at all important	○	○	◉	○	○	essential

Clear selection

Does your paper address subitem 11a-ii?

Copy and paste relevant sections from the manuscript (include quotes in quotation marks "like this" to indicate direct quotes from your manuscript), or elaborate on this item by providing additional information not in the ms, or briefly explain why the item is not applicable/relevant for your study

This is a protocol manuscript, discussion of this will be included in the outcome paper.

11b) If relevant, description of the similarity of interventions

(this item is usually not relevant for ehealth trials as it refers to similarity of a placebo or sham intervention to a active medication/intervention)

Does your paper address CONSORT subitem 11b? *

Copy and paste relevant sections from the manuscript (include quotes in quotation marks "like this" to indicate direct quotes from your manuscript), or elaborate on this item by providing additional information not in the ms, or briefly explain why the item is not applicable/relevant for your study

"Control group participants will be referred to attend the standard NHS-commissioned DSMES course that is delivered in their local area and be offered the choice of attending F2F or online delivery where both delivery methods are available. DSMES courses are a core NHS service with management guidelines recommending that all people with T2D attend a DSMES course (REF) and referral is incentivised in primary care (REF). The content and structure of DSMES courses are guided by a quality framework, requiring courses to be group-based, delivering an evidence-based curriculum to support self-management skills, and be delivered by skilled, competent staff (REF). A range of courses are delivered in the NHS, typically providing 6-14 hours of group-based education and support; these courses are typically based on a standardised curriculum that does not include culturally tailored information or advice. HEAL-D has been developed to align with the quality standards for DSMES programmes but is different to existing NHS courses in several ways. Principally, HEAL-D has been developed through a rigorous co-design process to reflect African and Caribbean cultural health beliefs and practices (REFS), providing tailored information, advice and support through culturally sensitive resources. Furthermore, delivery is led by a combination of healthcare professionals (dietitians), culturally concordant 'lay' facilitators and exercise trainers, and the programme includes participatory physical activity classes and practical cooking workshops. "

12a) Statistical methods used to compare groups for primary and secondary outcomes
NPT: When applicable, details of whether and how the clustering by care providers or centers was addressed

Does your paper address CONSORT subitem 12a? *

Copy and paste relevant sections from the manuscript (include quotes in quotation marks "like this" to indicate direct quotes from your manuscript), or elaborate on this item by providing additional information not in the ms, or briefly explain why the item is not applicable/relevant for your study

"Primary analysis of change in HbA1c at 12 months will be conducted using a mixed effects model with a random effect for the group attended, individuals in the control arm will be treated as groups of size 1 (47). Treatment arm, centre and baseline HbA1C will be included as fixed effects. The primary analysis will be repeated to include an interaction term between treatment and mode of delivery (F2F or online) to explore any differential treatment effect among groups. The presence of an interaction will be tested using a likelihood ratio test. The pre-specified subgroup analyses of the primary outcome will assess whether the effectiveness of the intervention is dependent on baseline HbA1c or centre. This will be assessed by adding interaction terms between group allocation and the potential effect modifiers to the linear regression, one at a time. The analysis exploring the change in treatment effect over time for the secondary outcomes will follow the methodology of the primary analysis, with an additional random effect for individual to account for within-individual correlation over time, a fixed effect for time and an interaction between treatment and time effect. Mediation analysis will be conducted using multilevel structural equation modelling (48). To account for missing data, multiple imputation will be undertaken, provided we have strong predictors of missingness and an appropriate imputation model. Diagnostic checks will be performed to assess this. The missing data mechanism will be assumed to be missing at random. Individual analyses on each imputed dataset will be combined using Rubin's rules. Sensitivity analyses will be conducted to assess the robustness of the primary conclusions to the imputation strategy used."

12a-i) Imputation techniques to deal with attrition / missing values

Imputation techniques to deal with attrition / missing values: Not all participants will use the intervention/comparator as intended and attrition is typically high in ehealth trials. Specify how participants who did not use the application or dropped out from the trial were treated in the statistical analysis (a complete case analysis is strongly discouraged, and simple imputation techniques such as LOCF may also be problematic [4]).

	1	2	3	4	5	
subitem not at all important	○	○	◉	○	○	essential

Clear selection

Does your paper address subitem 12a-i? *

Copy and paste relevant sections from the manuscript (include quotes in quotation marks "like this" to indicate direct quotes from your manuscript), or elaborate on this item by providing additional information not in the ms, or briefly explain why the item is not applicable/relevant for your study

"Primary analysis of change in HbA1c at 12 months will be conducted using a mixed effects model with a random effect for the group attended, individuals in the control arm will be treated as groups of size 1 (47). Treatment arm, centre and baseline HbA1C will be included as fixed effects. The primary analysis will be repeated to include an interaction term between treatment and mode of delivery (F2F or online) to explore any differential treatment effect among groups. The presence of an interaction will be tested using a likelihood ratio test. The pre-specified subgroup analyses of the primary outcome will assess whether the effectiveness of the intervention is dependent on baseline HbA1c or centre. This will be assessed by adding interaction terms between group allocation and the potential effect modifiers to the linear regression, one at a time. The analysis exploring the change in treatment effect over time for the secondary outcomes will follow the methodology of the primary analysis, with an additional random effect for individual to account for within-individual correlation over time, a fixed effect for time and an interaction between treatment and time effect. Mediation analysis will be conducted using multilevel structural equation modelling (48). To account for missing data, multiple imputation will be undertaken, provided we have strong predictors of missingness and an appropriate imputation model. Diagnostic checks will be performed to assess this. The missing data mechanism will be assumed to be missing at random. Individual analyses on each imputed dataset will be combined using Rubin’s rules. Sensitivity analyses will be conducted to assess the robustness of the primary conclusions to the imputation strategy used."

12b) Methods for additional analyses, such as subgroup analyses and adjusted analyses

Does your paper address CONSORT subitem 12b? *

Copy and paste relevant sections from the manuscript (include quotes in quotation marks "like this" to indicate direct quotes from your manuscript), or elaborate on this item by providing additional information not in the ms, or briefly explain why the item is not applicable/relevant for your study

"Primary analysis of change in HbA1c at 12 months will be conducted using a mixed effects model with a random effect for the group attended, individuals in the control arm will be treated as groups of size 1 (47). Treatment arm, centre and baseline HbA1C will be included as fixed effects. The primary analysis will be repeated to include an interaction term between treatment and mode of delivery (F2F or online) to explore any differential treatment effect among groups. The presence of an interaction will be tested using a likelihood ratio test. The pre-specified subgroup analyses of the primary outcome will assess whether the effectiveness of the intervention is dependent on baseline HbA1c or centre. This will be assessed by adding interaction terms between group allocation and the potential effect modifiers to the linear regression, one at a time. The analysis exploring the change in treatment effect over time for the secondary outcomes will follow the methodology of the primary analysis, with an additional random effect for individual to account for within-individual correlation over time, a fixed effect for time and an interaction between treatment and time effect. Mediation analysis will be conducted using multilevel structural equation modelling (48). To account for missing data, multiple imputation will be undertaken, provided we have strong predictors of missingness and an appropriate imputation model. Diagnostic checks will be performed to assess this. The missing data mechanism will be assumed to be missing at random. Individual analyses on each imputed dataset will be combined using Rubin’s rules. Sensitivity analyses will be conducted to assess the robustness of the primary conclusions to the imputation strategy used."

X26) REB/IRB Approval and Ethical Considerations [recommended as subheading under "Methods"] (not a CONSORT item)

X26-i) Comment on ethics committee approval

	1	2	3	4	5	
subitem not at all important	○	○	○	◉	○	essential

Clear selection

Does your paper address subitem X26-i?

Copy and paste relevant sections from the manuscript (include quotes in quotation marks "like this" to indicate direct quotes from your manuscript), or elaborate on this item by providing additional information not in the ms, or briefly explain why the item is not applicable/relevant for your study

"Human subject ethics review approvals: the trial has been approved by the Health Research Authority (IRAS ID: 326064) and East Midlands – Leicester South Research Ethics Committee (REC reference: 24/EM/0079), The trial will be conducted in full conformity with the current revision of the Declaration of Helsinki (last amended October 2000, with additional footnotes added 2002 and 2004), the UK Policy Framework for Health and Social Care Research (2017), and ICH-GCP relevant regulations.
Informed consent: potential participants who express an interest in participating in the trial will be provided with an information video and/or leaflet providing full details of the trial and what is entailed. Participants will be given an opportunity to discuss participation with a trial coordinator. Those who decide to participate will be required to provide written informed consent prior to the collection of research data; consent will be collected by trained research staff at the baseline assessment visit."

x26-ii) Outline informed consent procedures

Outline informed consent procedures e.g., if consent was obtained offline or online (how? Checkbox, etc.?), and what information was provided (see 4a-ii). See [6] for some items to be included in informed consent documents.

	1	2	3	4	5	
subitem not at all important	○	○	○	◉	○	essential

Clear selection

Does your paper address subitem X26-ii?

Copy and paste relevant sections from the manuscript (include quotes in quotation marks "like this" to indicate direct quotes from your manuscript), or elaborate on this item by providing additional information not in the ms, or briefly explain why the item is not applicable/relevant for your study

"Human subject ethics review approvals: the trial has been approved by the Health Research Authority (IRAS ID: 326064) and East Midlands – Leicester South Research Ethics Committee (REC reference: 24/EM/0079), The trial will be conducted in full conformity with the current revision of the Declaration of Helsinki (last amended October 2000, with additional footnotes added 2002 and 2004), the UK Policy Framework for Health and Social Care Research (2017), and ICH-GCP relevant regulations.
Informed consent: potential participants who express an interest in participating in the trial will be provided with an information video and/or leaflet providing full details of the trial and what is entailed. Participants will be given an opportunity to discuss participation with a trial coordinator. Those who decide to participate will be required to provide written informed consent prior to the collection of research data; consent will be collected by trained research staff at the baseline assessment visit."

X26-iii) Safety and security procedures

Safety and security procedures, incl. privacy considerations, and any steps taken to reduce the likelihood or detection of harm (e.g., education and training, availability of a hotline)

	1	2	3	4	5	
subitem not at all important	○	○	○	◉	○	essential

Clear selection

Does your paper address subitem X26-iii?

Copy and paste relevant sections from the manuscript (include quotes in quotation marks "like this" to indicate direct quotes from your manuscript), or elaborate on this item by providing additional information not in the ms, or briefly explain why the item is not applicable/relevant for your study

"Privacy and confidentiality: participant data will be pseudo-anonymised and treated in confidence and in compliance with International Council for Harmonisation of Technical Requirements for Pharmaceuticals for Human Use Good Clinical Practice (ICH-GCP), the UK Policy Framework for Health and Social Care and the UK General Data Protection Regulation (GDPR). All investigators and trial site staff will comply with the requirements of the Data Protection Act/GDPR with regards to the collection, storage, processing and disclosure of personal information and will uphold the Act’s core principles. The chief investigator (CI) will have access to the trial documentation and will be the data custodian."

RESULTS

13a) For each group, the numbers of participants who were randomly assigned, received intended treatment, and were analysed for the primary outcome

NPT: The number of care providers or centers performing the intervention in each group and the number of patients treated by each care provider in each center

Does your paper address CONSORT subitem 13a? *

Copy and paste relevant sections from the manuscript (include quotes in quotation marks "like this" to indicate direct quotes from your manuscript), or elaborate on this item by providing additional information not in the ms, or briefly explain why the item is not applicable/relevant for your study

This is a protocol manuscript, this information is not available to report at this stage.

13b) For each group, losses and exclusions after randomisation, together with reasons

Does your paper address CONSORT subitem 13b? (NOTE: Preferably, this is shown in a CONSORT flow diagram) *

Copy and paste relevant sections from the manuscript (include quotes in quotation marks "like this" to indicate direct quotes from your manuscript), or elaborate on this item by providing additional information not in the ms, or briefly explain why the item is not applicable/relevant for your study

This is a protocol manuscript, this information is not available to report at this stage.

13b-i) Attrition diagram

Strongly recommended: An attrition diagram (e.g., proportion of participants still logging in or using the intervention/comparator in each group plotted over time, similar to a survival curve) or other figures or tables demonstrating usage/dose/engagement.

	1	2	3	4	5	
subitem not at all important	(●)	○	○	○	○	essential

Clear selection

Does your paper address subitem 13b-i?

Copy and paste relevant sections from the manuscript or cite the figure number if applicable (include quotes in quotation marks "like this" to indicate direct quotes from your manuscript), or elaborate on this item by providing additional information not in the ms, or briefly explain why the item is not applicable/relevant for your study

This is a protocol manuscript, this information is not available to report at this stage.

14a) Dates defining the periods of recruitment and follow-up

Does your paper address CONSORT subitem 14a? *

Copy and paste relevant sections from the manuscript (include quotes in quotation marks "like this" to indicate direct quotes from your manuscript), or elaborate on this item by providing additional information not in the ms, or briefly explain why the item is not applicable/relevant for your study

"Recruitment commenced in August 2024 and is due to run over 11 months. "

14a-i) Indicate if critical "secular events" fell into the study period

Indicate if critical "secular events" fell into the study period, e.g., significant changes in Internet resources available or "changes in computer hardware or Internet delivery resources"

	1	2	3	4	5	
subitem not at all important	(●)	○	○	○	○	essential

Clear selection

Does your paper address subitem 14a-i?

Copy and paste relevant sections from the manuscript (include quotes in quotation marks "like this" to indicate direct quotes from your manuscript), or elaborate on this item by providing additional information not in the ms, or briefly explain why the item is not applicable/relevant for your study

This is a protocol manuscript, this information is not available to report at this stage.

14b) Why the trial ended or was stopped (early)

Does your paper address CONSORT subitem 14b? *

Copy and paste relevant sections from the manuscript (include quotes in quotation marks "like this" to indicate direct quotes from your manuscript), or elaborate on this item by providing additional information not in the ms, or briefly explain why the item is not applicable/relevant for your study

This is a protocol manuscript, this information is not available to report at this stage.

15) A table showing baseline demographic and clinical characteristics for each group

NPT: When applicable, a description of care providers (case volume, qualification, expertise, etc.) and centers (volume) in each group

Does your paper address CONSORT subitem 15? *

Copy and paste relevant sections from the manuscript (include quotes in quotation marks "like this" to indicate direct quotes from your manuscript), or elaborate on this item by providing additional information not in the ms, or briefly explain why the item is not applicable/relevant for your study

This is a protocol manuscript, this information is not available to report at this stage.

15-i) Report demographics associated with digital divide issues

In ehealth trials it is particularly important to report demographics associated with digital divide issues, such as age, education, gender, social-economic status, computer/Internet/ehealth literacy of the participants, if known.

	1	2	3	4	5	
subitem not at all important	(selected)	○	○	○	○	essential

Clear selection

Does your paper address subitem 15-i? *

Copy and paste relevant sections from the manuscript (include quotes in quotation marks "like this" to indicate direct quotes from your manuscript), or elaborate on this item by providing additional information not in the ms, or briefly explain why the item is not applicable/relevant for your study

This is a protocol manuscript, this information is not available to report at this stage.

16) For each group, number of participants (denominator) included in each analysis and whether the analysis was by original assigned groups

16-i) Report multiple “denominators” and provide definitions

Report multiple “denominators” and provide definitions: Report N’s (and effect sizes) “across a range of study participation [and use] thresholds” [1], e.g., N exposed, N consented, N used more than x times, N used more than y weeks, N participants “used” the intervention/comparator at specific pre-defined time points of interest (in absolute and relative numbers per group). Always clearly define “use” of the intervention.

	1	2	3	4	5	
subitem not at all important	(•)	( )	( )	( )	( )	essential

Clear selection

Does your paper address subitem 16-i? *

Copy and paste relevant sections from the manuscript (include quotes in quotation marks "like this" to indicate direct quotes from your manuscript), or elaborate on this item by providing additional information not in the ms, or briefly explain why the item is not applicable/relevant for your study

This is a protocol manuscript, this information is not available to report at this stage.

16-ii) Primary analysis should be intent-to-treat

Primary analysis should be intent-to-treat, secondary analyses could include comparing only “users”, with the appropriate caveats that this is no longer a randomized sample (see 18-i).

	1	2	3	4	5	
subitem not at all important	◉	○	○	○	○	essential

Clear selection

Does your paper address subitem 16-ii?

Copy and paste relevant sections from the manuscript (include quotes in quotation marks "like this" to indicate direct quotes from your manuscript), or elaborate on this item by providing additional information not in the ms, or briefly explain why the item is not applicable/relevant for your study

This is a protocol manuscript, this information is not available to report at this stage.

17a) For each primary and secondary outcome, results for each group, and the estimated effect size and its precision (such as 95% confidence interval)

Does your paper address CONSORT subitem 17a? *

Copy and paste relevant sections from the manuscript (include quotes in quotation marks "like this" to indicate direct quotes from your manuscript), or elaborate on this item by providing additional information not in the ms, or briefly explain why the item is not applicable/relevant for your study

This is a protocol manuscript, this information is not available to report at this stage.

17a-i) Presentation of process outcomes such as metrics of use and intensity of use

In addition to primary/secondary (clinical) outcomes, the presentation of process outcomes such as metrics of use and intensity of use (dose, exposure) and their operational definitions is critical. This does not only refer to metrics of attrition (13-b) (often a binary variable), but also to more continuous exposure metrics such as “average session length”. These must be accompanied by a technical description how a metric like a “session” is defined (e.g., timeout after idle time) [1] (report under item 6a).

	1	2	3	4	5	
subitem not at all important	(selected)	○	○	○	○	essential

Clear selection

Does your paper address subitem 17a-i?

Copy and paste relevant sections from the manuscript (include quotes in quotation marks "like this" to indicate direct quotes from your manuscript), or elaborate on this item by providing additional information not in the ms, or briefly explain why the item is not applicable/relevant for your study

This is a protocol manuscript, this information is not available to report at this stage.

17b) For binary outcomes, presentation of both absolute and relative effect sizes is recommended

Does your paper address CONSORT subitem 17b? *

Copy and paste relevant sections from the manuscript (include quotes in quotation marks "like this" to indicate direct quotes from your manuscript), or elaborate on this item by providing additional information not in the ms, or briefly explain why the item is not applicable/relevant for your study

This is a protocol manuscript, this information is not available to report at this stage.

18) Results of any other analyses performed, including subgroup analyses and adjusted analyses, distinguishing pre-specified from exploratory

Does your paper address CONSORT subitem 18? *

Copy and paste relevant sections from the manuscript (include quotes in quotation marks "like this" to indicate direct quotes from your manuscript), or elaborate on this item by providing additional information not in the ms, or briefly explain why the item is not applicable/relevant for your study

This is a protocol manuscript, this information is not available to report at this stage.

18-i) Subgroup analysis of comparing only users

A subgroup analysis of comparing only users is not uncommon in ehealth trials, but if done, it must be stressed that this is a self-selected sample and no longer an unbiased sample from a randomized trial (see 16-iii).

	1	2	3	4	5	
subitem not at all important	(selected)	○	○	○	○	essential

Clear selection

Does your paper address subitem 18-i?

Copy and paste relevant sections from the manuscript (include quotes in quotation marks "like this" to indicate direct quotes from your manuscript), or elaborate on this item by providing additional information not in the ms, or briefly explain why the item is not applicable/relevant for your study

This is a protocol manuscript, this information is not available to report at this stage.

19) All important harms or unintended effects in each group
(for specific guidance see CONSORT for harms)

Does your paper address CONSORT subitem 19? *

Copy and paste relevant sections from the manuscript (include quotes in quotation marks "like this" to indicate direct quotes from your manuscript), or elaborate on this item by providing additional information not in the ms, or briefly explain why the item is not applicable/relevant for your study

This is a protocol manuscript, this information is not available to report at this stage.

19-i) Include privacy breaches, technical problems

Include privacy breaches, technical problems. This does not only include physical "harm" to participants, but also incidents such as perceived or real privacy breaches [1], technical problems, and other unexpected/unintended incidents. "Unintended effects" also includes unintended positive effects [2].

	1	2	3	4	5	
subitem not at all important	◉	○	○	○	○	essential

Clear selection

Does your paper address subitem 19-i?

Copy and paste relevant sections from the manuscript (include quotes in quotation marks "like this" to indicate direct quotes from your manuscript), or elaborate on this item by providing additional information not in the ms, or briefly explain why the item is not applicable/relevant for your study

This is a protocol manuscript, this information is not available to report at this stage.

19-ii) Include qualitative feedback from participants or observations from staff/researchers

Include qualitative feedback from participants or observations from staff/researchers, if available, on strengths and shortcomings of the application, especially if they point to unintended/unexpected effects or uses. This includes (if available) reasons for why people did or did not use the application as intended by the developers.

	1	2	3	4	5	
subitem not at all important	(•)	( )	( )	( )	( )	essential

Clear selection

Does your paper address subitem 19-ii?

Copy and paste relevant sections from the manuscript (include quotes in quotation marks "like this" to indicate direct quotes from your manuscript), or elaborate on this item by providing additional information not in the ms, or briefly explain why the item is not applicable/relevant for your study

This is a protocol manuscript, this information is not available to report at this stage.

DISCUSSION

22) Interpretation consistent with results, balancing benefits and harms, and considering other relevant evidence

NPT: In addition, take into account the choice of the comparator, lack of or partial blinding, and unequal expertise of care providers or centers in each group

22-i) Restate study questions and summarize the answers suggested by the data, starting with primary outcomes and process outcomes (use)

Restate study questions and summarize the answers suggested by the data, starting with primary outcomes and process outcomes (use).

	1	2	3	4	5	
subitem not at all important	(selected)	○	○	○	○	essential

Clear selection

Does your paper address subitem 22-i? *

Copy and paste relevant sections from the manuscript (include quotes in quotation marks "like this" to indicate direct quotes from your manuscript), or elaborate on this item by providing additional information not in the ms, or briefly explain why the item is not applicable/relevant for your study

This is a protocol manuscript, this information is not available to report at this stage.

22-ii) Highlight unanswered new questions, suggest future research

Highlight unanswered new questions, suggest future research.

	1	2	3	4	5	
subitem not at all important	(selected)	○	○	○	○	essential

Clear selection

Does your paper address subitem 22-ii?

Copy and paste relevant sections from the manuscript (include quotes in quotation marks "like this" to indicate direct quotes from your manuscript), or elaborate on this item by providing additional information not in the ms, or briefly explain why the item is not applicable/relevant for your study

This is a protocol manuscript, this information is not available to report at this stage.

20) Trial limitations, addressing sources of potential bias, imprecision, and, if relevant, multiplicity of analyses

20-i) Typical limitations in ehealth trials

Typical limitations in ehealth trials: Participants in ehealth trials are rarely blinded. Ehealth trials often look at a multiplicity of outcomes, increasing risk for a Type I error. Discuss biases due to non-use of the intervention/usability issues, biases through informed consent procedures, unexpected events.

	1	2	3	4	5	
subitem not at all important	(•)	( )	( )	( )	( )	essential

Clear selection

Does your paper address subitem 20-i? *

Copy and paste relevant sections from the manuscript (include quotes in quotation marks "like this" to indicate direct quotes from your manuscript), or elaborate on this item by providing additional information not in the ms, or briefly explain why the item is not applicable/relevant for your study

This is a protocol manuscript, this information is not available to report at this stage.

21) Generalisability (external validity, applicability) of the trial findings

NPT: External validity of the trial findings according to the intervention, comparators, patients, and care providers or centers involved in the trial

21-i) Generalizability to other populations

Generalizability to other populations: In particular, discuss generalizability to a general Internet population, outside of a RCT setting, and general patient population, including applicability of the study results for other organizations

	1	2	3	4	5	
subitem not at all important	(●)	○	○	○	○	essential

Clear selection

Does your paper address subitem 21-i?

Copy and paste relevant sections from the manuscript (include quotes in quotation marks "like this" to indicate direct quotes from your manuscript), or elaborate on this item by providing additional information not in the ms, or briefly explain why the item is not applicable/relevant for your study

This is a protocol manuscript, this information is not available to report at this stage.

21-ii) Discuss if there were elements in the RCT that would be different in a routine application setting

Discuss if there were elements in the RCT that would be different in a routine application setting (e.g., prompts/reminders, more human involvement, training sessions or other co-interventions) and what impact the omission of these elements could have on use, adoption, or outcomes if the intervention is applied outside of a RCT setting.

	1	2	3	4	5	
subitem not at all important	(●)	○	○	○	○	essential

Clear selection

Does your paper address subitem 21-ii?

Copy and paste relevant sections from the manuscript (include quotes in quotation marks "like this" to indicate direct quotes from your manuscript), or elaborate on this item by providing additional information not in the ms, or briefly explain why the item is not applicable/relevant for your study

This is a protocol manuscript, this information is not available to report at this stage.

OTHER INFORMATION

23) Registration number and name of trial registry

Does your paper address CONSORT subitem 23? *

Copy and paste relevant sections from the manuscript (include quotes in quotation marks "like this" to indicate direct quotes from your manuscript), or elaborate on this item by providing additional information not in the ms, or briefly explain why the item is not applicable/relevant for your study

Trial registration
ISRCTN1434448 (24/05/2024; https://www.isrctn.com/ISRCTN14344948).

24) Where the full trial protocol can be accessed, if available

Does your paper address CONSORT subitem 24? *

Cite a Multimedia Appendix, other reference, or copy and paste relevant sections from the manuscript (include quotes in quotation marks "like this" to indicate direct quotes from your manuscript), or elaborate on this item by providing additional information not in the ms, or briefly explain why the item is not applicable/relevant for your study

This is the protocol manuscript.

25) Sources of funding and other support (such as supply of drugs), role of funders

Does your paper address CONSORT subitem 25? *

Copy and paste relevant sections from the manuscript (include quotes in quotation marks "like this" to indicate direct quotes from your manuscript), or elaborate on this item by providing additional information not in the ms, or briefly explain why the item is not applicable/relevant for your study

The trial is funded by the National Institute for Health Research (NIHR) Health Technology Assessment (HTA) Programme (NIHR151372) and is registered with ISRCTN (14344948), which adheres to the World Health Organization (WHO) Trial Registration Data Set.

X27) Conflicts of Interest (not a CONSORT item)

X27-i) State the relation of the study team towards the system being evaluated

In addition to the usual declaration of interests (financial or otherwise), also state the relation of the study team towards the system being evaluated, i.e., state if the authors/evaluators are distinct from or identical with the developers/sponsors of the intervention.

	1	2	3	4	5	
subitem not at all important	○	○	◉	○	○	essential

Clear selection

Does your paper address subitem X27-i?

Copy and paste relevant sections from the manuscript (include quotes in quotation marks "like this" to indicate direct quotes from your manuscript), or elaborate on this item by providing additional information not in the ms, or briefly explain why the item is not applicable/relevant for your study

This is a protocol manuscript, this information is not available to report at this stage.

About the CONSORT EHEALTH checklist

As a result of using this checklist, did you make changes in your manuscript? *

- ○ yes, major changes
- ◉ yes, minor changes
- ○ no

What were the most important changes you made as a result of using this checklist?

Using the checklist definitions/terminology, so instead of labelling the intervention delivery as 'online' or 'virtual', we have labelled it as 'video conferencing'

How much time did you spend on going through the checklist INCLUDING making changes in your manuscript *

Small amount of time making changes, but very time consuming to complete the checklist, providing all the quotes from the manuscript.

As a result of using this checklist, do you think your manuscript has improved? *

- ◉ yes
- ○ no
- ○ Other:

Would you like to become involved in the CONSORT EHEALTH group?

This would involve for example becoming involved in participating in a workshop and writing an "Explanation and Elaboration" document

- ○ yes
- ◉ no
- ○ Other:

Clear selection

Any other comments or questions on CONSORT EHEALTH

It takes too long to complete the questions in this form, it is easier with a checklist where you simply indicate page numbers. It would also be helpful to have a truncated questionnaire for protocol manuscripts.

STOP - Save this form as PDF before you click submit

To generate a record that you filled in this form, we recommend to generate a PDF of this page (on a Mac, simply select "print" and then select "print as PDF") before you submit it.

When you submit your (revised) paper to JMIR, please upload the PDF as supplementary file.

Don't worry if some text in the textboxes is cut off, as we still have the complete information in our database. Thank you!

Final step: Click submit !

Click submit so we have your answers in our database!

Submit Clear form

Never submit passwords through Google Forms.

This content is neither created nor endorsed by Google. - Terms of Service - Privacy Policy

Does this form look suspicious? Report

Google Forms
