# Supplementary material for: Culturally Tailored Diabetes Self-Management Education and Support Programs in Black African and Caribbean Adults With Type 2 Diabetes (HEAL-D): Protocol for a Multicenter, Pragmatic Randomized Controlled Trial
Source: JMIR Res Protoc. 2025 Sep 30;14:e71861. doi: 10.2196/71861 (PMC12521847; doi:10.2196/71861)
Supplement: Multimedia Appendix 2 [file resprot_v14i1e71861_app2.pdf]

Reviewer 1 Comments
Strength of this study :
The study has good primary and secondary outcome measures.
It is for benefit of patients and will reduce health inequality and cost to NHS.
It is a study that is very much needed and has resounding support from people with lived experience, PPIE and commissioners of NHS services.
Plain English summary is good with discreet headings for ease of reading without jargon and technical terms.
Study has strong PPI and very good PPI strategy.
Weakness if any - following are very few minor weakness that can be addressed.
Further improvement in PPI can be achieved by ensuring that PPI and has cultural competency as this would inevitably enhance participants trust and improve engagement. The same applies for front facing study team.
Eligibility criteria - there are three exclusion criteria - pregnant women, English speaking and suitability for group education.
Pregnant women- It is not clear as to what the rationale is for excluding pregnant women. So clear stated rationale for excluding pregnant women should be requested. unless there is a risk that cannot be mitigated study should consider putting in measures such that BABC pregnant women are able to participate. BABC women in general face worse health inequality and discrimination. BABC pregnant women face even more discrimination via such exclusion. Hence every step should be taken to ensure that health inequality is not exasperated by research.
English speaking - this exclusion criteria can be mitigated and such mitigation should be considered and put in place so as not to increase health inequality.
suitability for group education - this criteria needs clear parameters firstly by defining what it means by suitability for group education, then setting clear checklist for recruiters to abide by otherwise there is a risk of bias and or discrimination because of individual perceptions and judgements.
The main purpose of this study is to reduce health inequality hence it feels missed opportunity when such small pockets of hard to reach patients are excluded.

Reviewer 2 Comments
Overall comments
This proposal aims to address a very important clinical and public health problem and seeks to understand how people from Black African and Black Caribbean communities in the UK can be empowered to self-manage T2D which occurs at a higher incidence and lower age compared to their White counterparts.
Applicants have addressed reviewers' points from Stage 1.
Mental health variables are very important in the context of obesity management and have been added as secondary outcomes to be measured using standardised and validated questionnaires.
The proposed statistical analysis appears robust.
The PPI strategy is comprehensive and well developed.

Major comments
Given that the uptake of such interventions is expected to be low in the population of interest (likely to live in deprived areas and have low digital literacy) and some people do not speak English, it is important to show that there is an effective method of reaching to such populations.
Applicants have provided the details about the facilitators and barriers demonstrated in the feasibility study "Retention in our feasibility trial (6-months follow-up) was 93%. Factors facilitating retention were financial incentives, trusted relations between the researchers and participants and an opportunity to have clinical measurements. The primary reason for loss to follow-up was lengthy overseas travel. We have drawn on these insights to maximise protocol compliance for our data collection at all timepoints."
What will be done if recruitment does not meet the expected targets?

Minor comments
The applicants should consider stating right at the beginning of the application the specific components of the intervention that apply to the cultural context in question and how it differs from the current standard of care which has a poor uptake among Black African and Black Caribbean people.
There seem to be too many abbreviations and it is not easy to read the long document.
Follow-up duration of 29 months seems like an arbitrary choice. How was this decision made?
There are subtitles "our aim" and "our research proposal" in the Plain English summary. The word our can be deleted as it is clear whose aim and research proposal is being presented.
Tanita does not measure body fat directly but rather, estimates it using bioimpedance. The applicants should replace the word measured with estimated and also clarify if they are referring to total body fat (as opposed to subcutaneous or visceral).
The applicants should consider the possibility of random and systematic error in measuring lifestyle variables (physical activity and diet). IPAQ has been proposed to measure physical activity. However, the applicants have not specified if this is going to be short or long form of the IPAQ. The cited validation paper by Craig et al. included 12 countries in this validation study and the UK was one of them with Cambridge and Bristol being study sites. This paper does not directly state the ethnic composition of the study population, but it is very likely that the participants in Cambridge and Bristol were predominantly of White Caucasian descent. They were also reported to be in good health and were well educated. This raises a question of whether this instrument would be valid and reliable in Black African and Black Caribbean participants as it may not capture culturally specific physical activities. Is there another validated self-reported instrument to measure physical activity in Black populations?
Applicants stated that pedometers will be given to participants to measure daily step count. What type of pedometer will be used and has it been validated against a superior criterion? How will step count data be analysed?
The applicants should consider stating if the £25 retail voucher is going to include the stores with access to fast food.
When people with T2D lose weight, their requirements for glucose-lowering therapy tends to decrease and they can be at risk of hypoglycaemia if on insulin or sulphonylurea. It is important to take this into

account. In the current version, hypoglycaemia does not seem to be mentioned.

Reviewer 3 Comments

This second stage application addresses an important topic in diabetes care in attempting to estimate the effectiveness of a culturally appropriate behavioural intervention in Black-African and Black-Caribbean people with type 2 diabetes. The application makes a strong case that this research is needed by the community for whom the intervention is designed and that the whole process of developing and evaluating the intervention has been very inclusive of this community. If the intervention were successful in achieving a statistically mean difference from baseline (compared to a standard self-management education and support programme) of 5 mmol/mol in HbA1c as the primary outcome, this would be clinically meaningful. Thus I would conclude that the proposed trial is addressing an important question and has the potential to make a difference to this under-served population of patients.

The full trial application has followed a staged process of development and initial testing including a randomised feasibility study that was published in Diabetes Open Research and Care in 2021. This very thorough feasibility study demonstrated the feasibility of recruiting, randomising and retaining participants, at least until the endpoint measurement point in the feasibility study which was at 6-8 months. The full trial endpoint measurement is proposed to be at 12 months, so there is a chance that retention might be lower than in the feasibility study which was shorter. The intervention was acceptable to patients and practitioners and was delivered with acceptable fidelity. The estimate of effect size in the feasibility study was 2.8 mmol/mol with a 95% CI of -9.5 to 3.9 mmol/mol. The full trial is powered to detect a minimal clinically important difference of 5 mmol/mol. The fact that the feasibility study had a lower mean effect size should not impact on the decision about whether the MCID proposed would be detectable in the full trial since the feasibility study was not powered to detect such an effect. The one concern that one might have is that the full trial design (following the first stage) allows for personal choice to determine whether the intervention is delivered face-to-face or remotely. As I understand it the feasibility study intervention delivery was entirely face-to-face. It is possible, but unknowable at this stage, that remote delivery might be as effective as face-to-face. The applicants have tested the acceptability of an online version of their intervention, which is commendable, but they haven't evaluated its effectiveness. If it is not effective, there is a risk that the effect of the intervention might be diluted by allowing both face-to-face and remote delivery. In turn this could mean that the trial as currently designed could fail to demonstrate an effect for the face-to-face element of the intervention which appeared to be promising in the feasibility study. It would be poor value for money if the study were to report an inconclusive result. I appreciate that the allocation to face-to-face or remote will not be randomised but will be a matter of personal choice. Thus it won't be possible to do a stratified analysis comparing the relative effectiveness of the F2F or remote intervention because there are likely to be individual differences that are related to which intervention delivery mode participants chose. However, clinically, I would expect there to be interest in this question especially if there is a non-significant overall effect with evidence of heterogeneity between the two delivery modes. Consideration about what would be possible to address this issue in an a priori statistical analysis plan would be sensible.

I did not see the first stage application but one of the questions raised was the cost of the project. £2.4 million for a trial of 500 participants with main outcome assessment at 12 months does seem extremely expensive, albeit accepting that the proposal includes other key elements such as various secondary outcomes, a cost-effectiveness analysis, process evaluation and an embedded mixed methods study. The applicants were clearly asked to reduce their costs and have reduced their request by £166k which represents a reduction of something around 7%. The current proposal only provides headline figures so as a reviewer there is no chance to delve into the details of the costing but I would have residual concerns about value for money.

Overall this is a trial that is addressing an important question and has been developed and tested in a sequential way including in a randomised feasibility study. The full trial includes appropriate stop-go considerations related to recruitment and I would be confident that it is feasible. The concern is that the inclusion of a remote delivery option might dilute the effect of the face-to-face intervention that was tested in the feasibility study and that the trial as designed might, as a consequence, find no overall significant effect and be under-powered to detect a clinically meaningful difference in the individuals who elected to have the face-to-face intervention. If this were the case with a budget of £2.4 million, that would represent poor value for money as one would be left with clinical uncertainty about effectiveness. In an ideal world, the trial would be made cheaper and more efficient and be powered to detect a difference both overall and in the two sub-groups which represent fundamentally different ways of delivering the intervention.

Reviewer 4 Comments

Thank you very much for letting me review this very interesting application.

The applicants propose to run a multicentre, pragmatic, randomised controlled trial comparing culturally tailored versus standard diabetes self-management education and support (DSMES) programmes in Black-African and Black-Caribbean (BABC) adults with type 2 Diabetes Mellitus to assess the relative effectiveness and cost-effectiveness of these two programmes.

My expertise is in health services research and health economics and my comments are related to these areas of expertise.

The interventions being compared are culturally tailored DSMES versus usual care DSMES:
- the study will recruit adults (18 years and over) of BABC ethnicity with Type 2 Diabetes. At time of recruitment potential participants will be given information about the study and the interventions being compared. Potential participants are not newly diagnosed individuals and might already have experienced standard care. These participants could see this intervention as ‘oh here we go again’, showing lack of interest compared with a culturally tailored programme. Wouldn’t be more appropriate to recruit newly diagnosed individuals with no previous experience on either of the interventions being compared?

- Covid-19 have lasting effects on the NHS health care. Would participants randomised to usual care have access to the DSMES programme at similar timing/delays to those randomised to the culturally tailored DSMES programme? While a pragmatic trial is expected to compare two alternative care pathways, the applicants should make sure differences in effectiveness (and cost-effectiveness) are explained by the differences in the programmes (tailored vs not tailored) and not due to differences in access to health care (timely vs. delayed).

The health economics component proposed a within RCT analysis, a modelling extrapolation and an economic evaluation of the implementation and scale up of the programme. This seems quite an undertaking for a 12-month FTE (i.e., 48-month project and 20% and 5%, respectively, for the two co-applicants leading the health economics component). This reviewer does not have access to the complete costing for the project to see if further resources were included for the health economics component of the project. The applicants should make sure the health economic component has been costed properly.

Within RCT economic evaluation:
- the relevant period for this analysis should be defined (is this 12 or 24 months?)
- It is not clear if baseline EQ-5D will be collected. Adjusting for baseline utility score is standard practice for within RCT cost-utility analysis.

Extrapolation beyond trial follow-up:
- The applicants propose to use the UKPDS-OM2 (applicants' reference 82) to extrapolate outcomes beyond the trial follow-up. Please note a recent study by Keng et al. (VALUE HEALTH. 2022; 25(3):435–442) demonstrating that "the UKPDS-OM2 overpredicts the risks of myocardial infarction, stroke, heart failure, and deaths over 10 years of follow up in A Study of Cardiovascular Events in Diabetes (ASCEND), one of the largest trials in people with diabetes in the United Kingdom that followed participants from 2005 to 2017. In particular, the performance of the UKPDS-OM2 was found to be poorer in older patients who received a diagnosis of diabetes at an older age". Also, the UKPDS risk prediction equations for complications were estimated for a cohort with 81% White and only 8% Afro-Caribbean individuals (Keng. et al. VALUE HEALTH. 2022; 25(3):435–442; Supplementary material, Appendix Table 4). The applicants should ensure the proposed economic analysis is appropriate to answer the question posed.

The applicants proposed to explore population heterogeneity (specifically associated with the presence of multiple long-term conditions -MLTC-). Is the trial sample size large enough to allow for this type of analysis given the number of possible MLTC?

Reviewer 5 Comments
DOCUMENTS PROVIDED

I have seen the summary application including
- Scientific abstract
- Plain English summary
- The Full Protocol

SUMMARY

- The HEAL-D self management programme for Type 2 diabetes in ethnic minority populations has been developed under an NIHR research programme and shown to be internally reliable with good patient acceptability and in principle effective.
- The trial proposed aims to answer a very important research question. This is to identify whether HEAL-D produces better outcomes for ethnic minority groups than conventional interventions for Type 2 Diabetes.
- The research proposal appears to have answered the questions outstanding from the stage 1 submission
- The systematic review of evidence the authors have conducted showed that of 14 RCTs examining culturally sensitive education programmes for diabetes 65 resulted in hba1c reductions. This may indicate that the manner in which trials are conducted and their size may affect outcomes. The proposed trial will be the first in the UK so it should be designed and powered to provide the highest quality result.

REPORT QUESTIONS
1. How will the research make a difference? In your experience, will the research, as described, produce or have the potential to lead to, findings that will enable change and benefit patients and the public? This change could impact on the public, patients, carers, health and social care practitioners, decision makers and providers of health and social care services.
This research may improve the healthcare for a population that do not respond well to conventional approaches of self-education for diabetes and are at high risk.
2. Is the proposed research feasible from your perspective? Can it be successfully delivered as described in the application? If not, please explain which areas would need to be addressed and why. You may want to consider the proposed study approach, the acceptability to all participants or any potential barriers to the research being successful.
The research is feasible and the timescale appropriate. I have not seen the detailed costs but for a trial of this duration and complexity they do not appear unreasonable.
3. What else could the applicants do to improve the research proposed?
See my Comments below.

COMMENTS

The manner in which the selection of patients will be conducted means that a pragmatic sample of volunteers who meet the criteria is to be included. This may result in bias arising from recruiting from an already motivated group. Does this limit the future applicability of the results to the general population, which may well include less motivated individuals? The authors should discuss this and describe how their recruitment maximises the value of HEAL-D for general implementation in the UK.

The patient level randomisation means that patients may be exposed to others in their DSMES provider or medical practice (primary or secondary) who have the opposite randomisation. This may dilute the effects of the intervention. The authors might comment on how far this has been taken into account in the study design and in the power calculation.

12 months follow-up for the primary outcome and 24 month follow-up for the secondary outcomes would appear inconsistent.

Changes in behaviour can take time to become embedded and the consequences of Type 2 diabetes are measured in years. I wonder therefore if the researchers would be advised to take a longer view about the time taken for a significant divergence of hba1c between the randomised groups (Primary outcome). Hba1c is usually measured 6 monthly in clinical practice as change is slow to take effect and consistent change requires prolonged assessment. Would the authors consider extending the Primary outcome to 24 months? Patients are in any case being followed for this period of time. I note the extension from 6 months (feasibility study) to 12 months but this is still short.

The outcomes are proxy markers in the sense that they indicate control and risk. The disease outcomes of, for example, retinopathy, nephropathy, foot disease and cardiovascular endpoints are not included. This is reasonable for a short trial. However, this further underlines the need for this trial to confirm that any changes in proxy markers are sustained.

An MCID of 5 mol/mol hba1c reduction appears very ambitious to me and I think the power calculation should be checked to ensure the recruitment of 300 patients is sufficient particularly as the intervention is effectively divided into two parts (face to face, and on line which might have different outcomes).

The extensive involvement of patients in developing the HEAL-D programme and in steering this pragmatic trial is described well and is should contribute strongly to the success of this project.

The assessment of multiple longterm conditions is not central to this trial and I wondered how it could be justified in terms of the generalisability of primary and secondary outcomes. While the data will be interesting I did wonder if this could be the subject of a different study.

The Cost effectiveness of the intervention will be crucial and I have struggled to see a detailed description of how this is to be carried out. I would expect- fully costed personnel costs with management overhead, estate and IT, impact on primary and community care services, etc vs. opportunity cost in reducing longterm disease outcomes in patients with type 2 diabetes.

Reviewer 6 Comments
This application is looking at the effectiveness and cost-effectiveness of culturally tailored versus standard diabetes self-management programmes in Black-African and Black-Caribbean adults with type 2 diabetes. My comments relate mainly to the methodological aspects of the trial.

Design – this is a single pragmatic two-arm trial of HEAL-D versus standard diabetes self-management programme, with participants choosing face to face or online attendance. I was unclear if this choice relates to the HEAL-D intervention only or also the standard care intervention (i.e. what is current NHS care for this population – is it face to face or online and is it group based on individual). I'd also recommend adding a pre-planned subgroup analysis to see if mode of delivery impact on outcome assessments, especially the primary outcome.

Internal pilot - there is a clear internal pilot based on number of site opening and number of participants recruited. This seems realistic based on the team's previous work as part of their feasibility study.

Recruitment – It's good to see letters of support from potential trial sites and shows engagement from the local community. The eligibility criteria is defined as adults who are English speaking, Black-African, Black Caribbean ethnicity. In terms of inclusively, I believe the team should also consider including non-English speaking participants and include the use of translation services where this is applicable. I may have missed it but I was unable to find details on how participants will be approached and consent into the trial; details are provided for consent as part of the process evaluation but I could not find the relevant information in relation to the actual trial.

Primary outcome – is change in HbA1c from baseline to 12 months. More detail is need here on how the primary outcome will be assessed and recorded, for example I was unclear whether the primary outcome as currently described is HbA1c at 12 month or over the 12 month period. Is this a one off measure at 12 months or repeated measured taken over the 12 month period. Follow up at 6, 12 and 24 months and will be collected at a 2 hour study site to a clinical research facility. This is pretty resource intensive and many of the patient reported outcomes could be collected electronically via questionnaires without the need for a pre-longed clinic visits. I would encourage the team to consider a more efficient and less resource intensive method data collection where possible.

Sample size and statistical analysis – 300 participants, assuming 90%, and 15% loss to follow up at 12 months. In the section of sample size the intervention is described as a group based intervention with groups of up to 12 participants when the intervention was delivered face to face and groups of up to 8

participants when the intervention was delivered online. I was unclear whether this relates to both the HEAL-D intervention and standard care or just HEAL-D (see earlier comment). The sample size is inflated by a small ICC of 0.01 but I did not fully understand the basis for 0.01 as the ICC and also justification for the group size. Very little data is provided on the plans for statistical analysis particularly in relation to the primary outcome.

Blinding – no information is provided on blinding of clinician assessed outcomes, which is particularly important for subjective outcomes.
